# Serum Biomarkers for Chronic Renal Failure Screening and Mechanistic Understanding: A Global LC-MS-Based Metabolomics Research

**DOI:** 10.1155/2022/7450977

**Published:** 2022-07-30

**Authors:** Xiaojuan Su, Ruru Ren, Lingling Yang, Chao Su, Yingli Wang, Jun Lu, Jing Liu, Rong Zong, Fangfang Lu, Gidion Wilson, Shuqin Ding, Xueqin Ma

**Affiliations:** ^1^Department of Pharmaceutical Analysis, School of Pharmacy, Ningxia Medical University, 1160 Shenli Street, Yinchuan 750004, China; ^2^Yinchuan Weikang Nephrology Hospital, Intersection of Lijing North Street and Agricultural Materials Lane, Yinchuan 750004, China; ^3^School of Clinical Medicine, Ningxia Medical University, 692 Shenli Street, Yinchuan 750004, China; ^4^Key Laboratory of Hui Ethnic Medicine Modernization, Ministry of Education, Ningxia Medical University, 1160 Shenli Street, Yinchuan 750004, China

## Abstract

Chronic kidney disease, including renal failure (RF), is a global public health problem. The clinical diagnosis mainly depends on the change of estimated glomerular filtration rate, which usually lags behind disease progression and likely has limited clinical utility for the early detection of this health problem. Now, we employed Q-Exactive HFX Orbitrap LC-MS/MS based metabolomics to reveal the metabolic profile and potential biomarkers for RF screening. 27 RF patients and 27 healthy controls were included as the testing groups, and comparative analysis of results using different techniques, such as multivariate pattern recognition and univariate statistical analysis, was applied to screen and elucidate the differential metabolites. The dot plots and receiver operating characteristics curves of identified different metabolites were established to discover the potential biomarkers of RF. The results exhibited a clear separation between the two groups, and a total of 216 different metabolites corresponding to 13 metabolic pathways were discovered to be associated with RF; and 44 metabolites showed high levels of sensitivity and specificity under curve values of close to 1, thus might be used as serum biomarkers for RF. In summary, for the first time, our untargeted metabolomics study revealed the distinct metabolic profile of RF, and 44 metabolites with high sensitivity and specificity were discovered, 3 of which have been reported and were consistent with our observations. The other metabolites were first reported by us. Our findings might provide a feasible diagnostic tool for identifying populations at risk for RF through detection of serum metabolites.

## 1. Introduction

Renal failure (RF), one of the main kidney diseases, is a major health problem throughout the world [1]. It is always associated with high cardiovascular morbidity and mortality, and the progression of RF to end-stage renal disease (ESRD) is believed to be closely related to the accumulation of metabolites in the blood [2, 3]. Once people suffer from RF, it can impose a huge financial burden on their families as it increases the need for costly renal replacement therapies (peritoneal dialysis, haemo-dialysis, and haemo-diafiltration) and kidney transplantation [4]. Although important features, such as dyslipidemia and catabolism, remain incompletely understood, small molecules related to impaired renal function are believed to contribute to mortality in RF patients. Moreover, decades of research have identified numerous small molecules as potential uremic toxins to exert detrimental activities [1]. At the early stages of RF, little signs or symptoms are obscure and often cannot be detected until the later stages. The risk of mortality is thus increased with the progression of RF. Hence, looking for more metabolite biomarkers with high sensitivity and specificity can provide more choices for the diagnosis of RF and give a better mechanistic understanding of this disease [3, 5, 6]. Although current clinical analytical methods are accurate in diagnosing advanced kidney dysfunction, they are not the case for the early stages [7], and most importantly, tools for predicting the risk of kidney disease towards RF are still lacking. Discovering and developing accurate biomarkers for the diagnosis of RF always means a huge challenge, and the interactions between metabolism and kidney disease are particularly complex [8]. The kidney organ performs the energy-intensive task of solute and water reabsorption from more than 100 L/day of filtrate, with distinct cell types exerting different metabolic functions in widely disparate oxygen tensions and osmotic environments across the nephron. Kidney function was related to approximately one-third of detected metabolites in both general populations and RF patients [8–10]. Decreased kidney function can directly affect the systemic metabolism, homeostasis of body fluids, electrolytes, acid-base equilibrium, bone metabolism, erythropoiesis, and blood coagulation [9, 11–15]. Previous studies employing ion-exchange chromatography or LC have identified several changes in amino acid metabolism in patients with ESRD. And recent developments in advanced metabolomics techniques allow the metabolic conditions to be evaluated sensitively and comprehensively [16].

Metabolomics, also known as metabonomic or metabolic profiling, has been proven to possess widespread applicability for screening clinical biomarkers. Given kidney organ is responsible for concentrating and excreting a variety of metabolites from body [17, 18], metabolomics method is particularly well suited for screening biomarkers of RF. According to the published data, several metabolite markers including dicarboxylic acids (adipate, malonate, methylmalonate, and maleate), biogenic amines, nucleotide derivatives, phenols, and sphingomyelins were identified in ESRD by applying LC-MS-based metabolomics technology [1]. Lactose, 2-O-glycerol-*α*-d-galactopyranoside, D-threitol and tyrosine were proven to be associated with the risk of ESRD [19]. Therefore, the more sensitive and specific biomarkers are identified, the more simply and easily the disease can be diagnosed. Meanwhile, besides the biomarkers themselves, metabolic fingerprints also provide insight into the complications and high mortality rates of RF, and potentially lead to the discovery of novel treatments [20]. Hence, in the present study, we employed a Q-Exactive HFX Orbitrap UHPLC-MS/MS-based metabolomics technology to elucidate the different metabolites in RF that could be further developed as potential biomarkers for its diagnosis and treatment.

## 2. Materials and Methods

### 2.1. Subjects and Reagents

27 serum samples were collected from patients with RF (RF), and corresponding healthy control serum were obtained from 27 healthy people (HC). All of the serum samples were obtained from Yinchuan Weike Renal Specialty Hospital, from the year 2018 to 2019. Detailed information of the serum samples are listed in Table 1 and Table 2, and the clinical characteristics of patients are presented in Supplementary Table S1. In this study, patients at CKD stage 5 with an estimated glomerular filtration rate (eGFR) < 15 mL/min/1.73 m^2^ (chronic kidney disease, CKD 5, *n* = 27) and HC participants without acute inflammatory disease were selected for screening serum biomarkers of chronic renal failure. The serum samples were packaged and stored at −80°C for later analysis. Methanol, acetonitrile, ammonium acetate, and ammonium hydroxide were all chromatography grade (CNW Technologies, Germany).

### 2.2. Metabolites Extraction

Experimental processions were outlined as follows: 50 *μ*L of each serum sample were transferred to an EP tube, and 200 *μ*L of extraction solution (methanol: acetonitrile = 1 : 1 (*v/v*), containing isotopically-labelled internal standard mixture were added. The internal standard in positive/negative ion mode is tmao-d9/hippuric acid-d5), sonicated for 10 min in an ice-water bath, and incubated at −40°C for 1 h to precipitate proteins. Then, the suspension was centrifuged at 12000 rpm at 4°C for 15 min, and the supernatant was transferred to a fresh glass vial for further analysis. Moreover, the quality control (QC) sample was prepared by mixing an equal aliquot of the supernatants from all of the samples.

### 2.3. Q-Exactive HFX Orbitrap LC-MS/MS Analysis

LC-MS/MS was performed using an UHPLC system (Vanquish, Thermo Fisher Scientific) coupled with a Q-Exactive HFX mass spectrometer (Orbitrap MS, Thermo Fisher Scientific) to acquire MS/MS spectra in an information-dependent acquisition mode in the control of the acquisition software (Xcalibur, Thermo Fisher Scientific). An UHPLC experiment was performed as described below: BEH amide column (2.1 mm × 100 mm, 1.7 *μ*m), a binary mobile phase consistent of water with 25 mmol/L ammonium acetate and 25 mmol/L ammonia hydroxide (pH 9.75) as mobile phase A, and acetonitrile as mobile phase B, using a gradient elution as follows: 0∼0.5 min, 95% B; 0.5∼7.0 min, 95%∼65% B; 7.0∼8.0 min, 65%∼40% B; 8.0∼9.0 min, 40% B; 9.0∼9.1 min, 40%∼95% B; 9.1∼12.0 min, 95% B. The column temperature was held at 30°C, while the autosampler temperature was maintained at 4°C, and the injection volume was 2 *μ*L. The ESI source conditions were set as follows: sheath gas flow rate was 50 arb, aux gas flow rate was 10 arb, capillary temperature was 320°C, full MS and MS/MS resolutions were acquired at 60000 and 7500, respectively; collision energy was set as 10/30/60 in normalized collision energy mode, spray voltage was 3.5 kV for positive mode and 3.2 kV for negative mode, respectively.

### 2.4. Data Preprocessing and Annotation

The raw data were first converted to mzXML format using ProteoWizard, and then processed with an in-house program, which was developed by using the R package with XCMS, for peak detection, extraction, alignment, and integration. Thirdly, an in-house MS2 database (BiotreeDB) was applied to metabolite annotation, with the cutoff at 0.3. To better analyze the data, a set of preparations and data management were performed based on the original peaks, which mainly included the following steps: (1) The noise was removed by filtering individual peaks. (2) Deviation values were filtered based on relative standard deviation (RSD) of 30%. The missing values recoding in the original peak area were simulated by 1/2 of the minimum values. (3) The peak area was normalized by an internal standard.

### 2.5. Statistical Analysis

Based on the normalized peak area, differential metabolites were identified by employing *t*-test with SPSS (25.0), principle component analysis (PCA) and orthogonal projections to structures-discriminant analysis (OPLS-DA) with SIMCA software (V16.0.2, Sartorius Stedim Data Analytics AB, Umea, Sweden). The variable importance in the projection (VIP) value for each metabolite was extracted from the OPLS-DA models, and the volcano plot was used to excavate the candidate metabolites in comparison groups. Metabolites with a VIP value greater than 1 and *p* value less than 0.05 were considered as significant differential biomarkers. Then, the hierarchical cluster analysis (HCA), KEGG annotation, metabolic pathway and random forest analysis on the potential differential metabolites were carried out. Finally, receiver operating characteristics (ROC) curves and dot plots based on the relative quantification of the differential metabolites were established.

## 3. Results

### 3.1. Quality Control Results

Quality control in two aspects of metabolites was performed, including instrument quality control (instrument stability and internal standard response) and data quality control (presentation of QC samples in PCA analysis). The results are shown in Supplementary Figure S1. The PCA results of the QC and test samples in both positive and negative ion modes indicated the stability of the instrument and the good quality of the data.

### 3.2. Metabolomic Outline of Renal Failure

**Figure 1:**
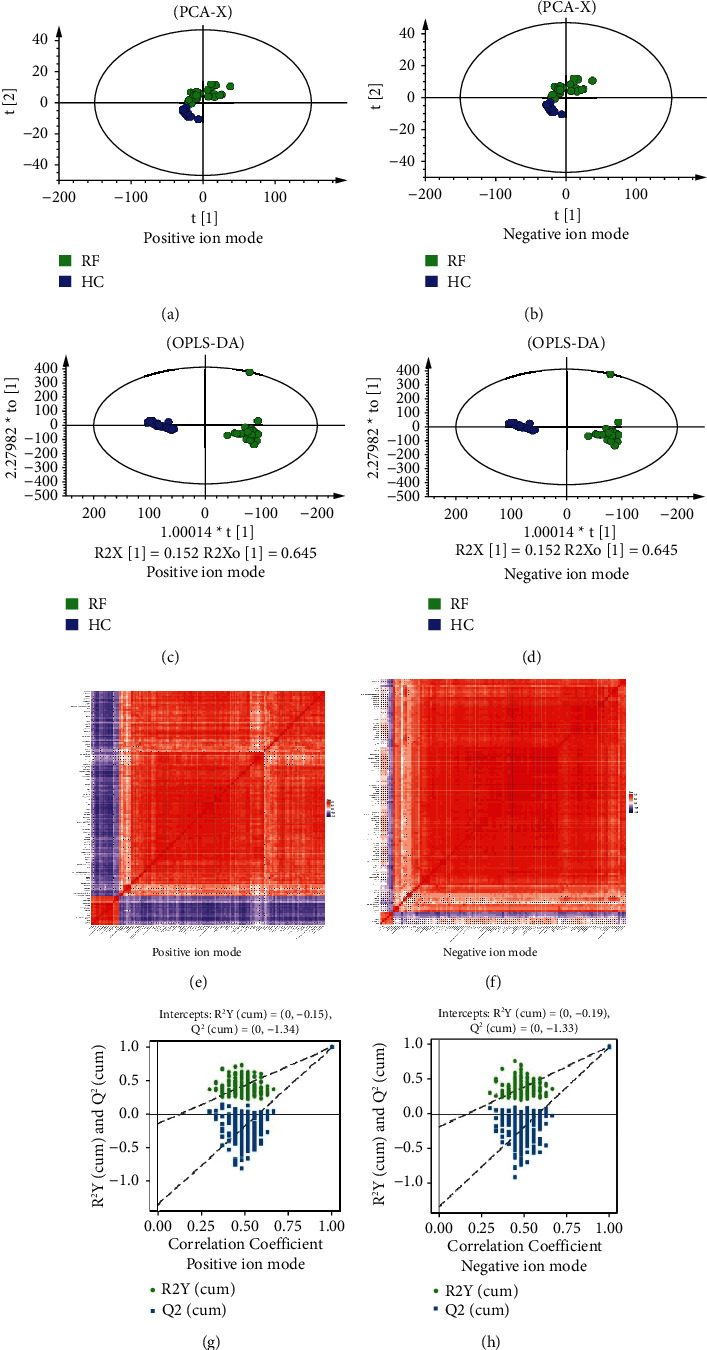
Score plots of PCA, OPLS-DA, correlation analysis and permutation test for group RF vs HC. (a) and (b) are PCA score plots in positive and negative modes, respectively; (c) and (d) are OPLS-DA score plots in positive and negative modes, respectively; (e) and (f) are correlation analysis heat map in positive and negative modes, respectively; (g) and (h) are permutation tests of OPLS-DA in positive and negative modes, respectively.

According to the above Q-Exactive HFX Orbitrap LC-MS/MS methods, a total of 8066 and 7050 peaks were extracted from positive (POS) and negative ion modes (NEG), respectively. Based on the above three data management steps, 5577 and 5042 peaks were preserved in POS and NEG modes, respectively. The results of the PCA, OPLS-DA and correlation analysis showed that RF and HC groups could be clearly differentiated (Figures 1(a)–1(f), the original figures with high pixels in Supplementary Figures S2A and S2B.). And the R^2^Y values of permutation test of the OPLS-DA model for RF and HC were 0.988 and 0.971 (Figures 1(g) and 1(h)), respectively, which implied that the original model had good robustness and there was no over-fitting phenomenon.

**Figure 2:**
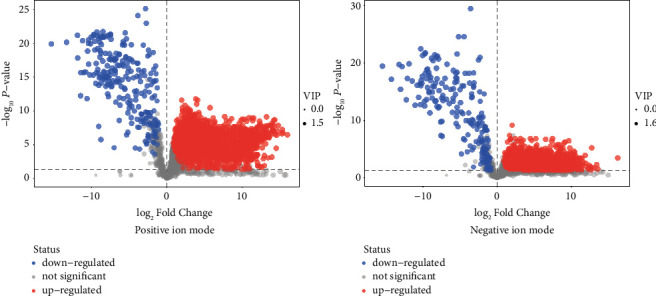
Volcano plot for group RF vs HC.

**Figure 3:**
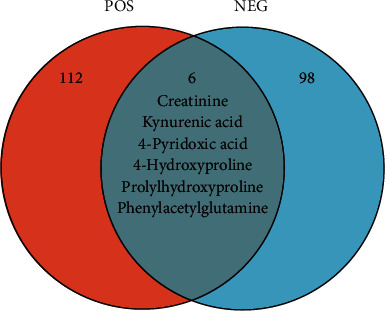
Differences in differential metabolites for POS vs NEG.

**Figure 4:**
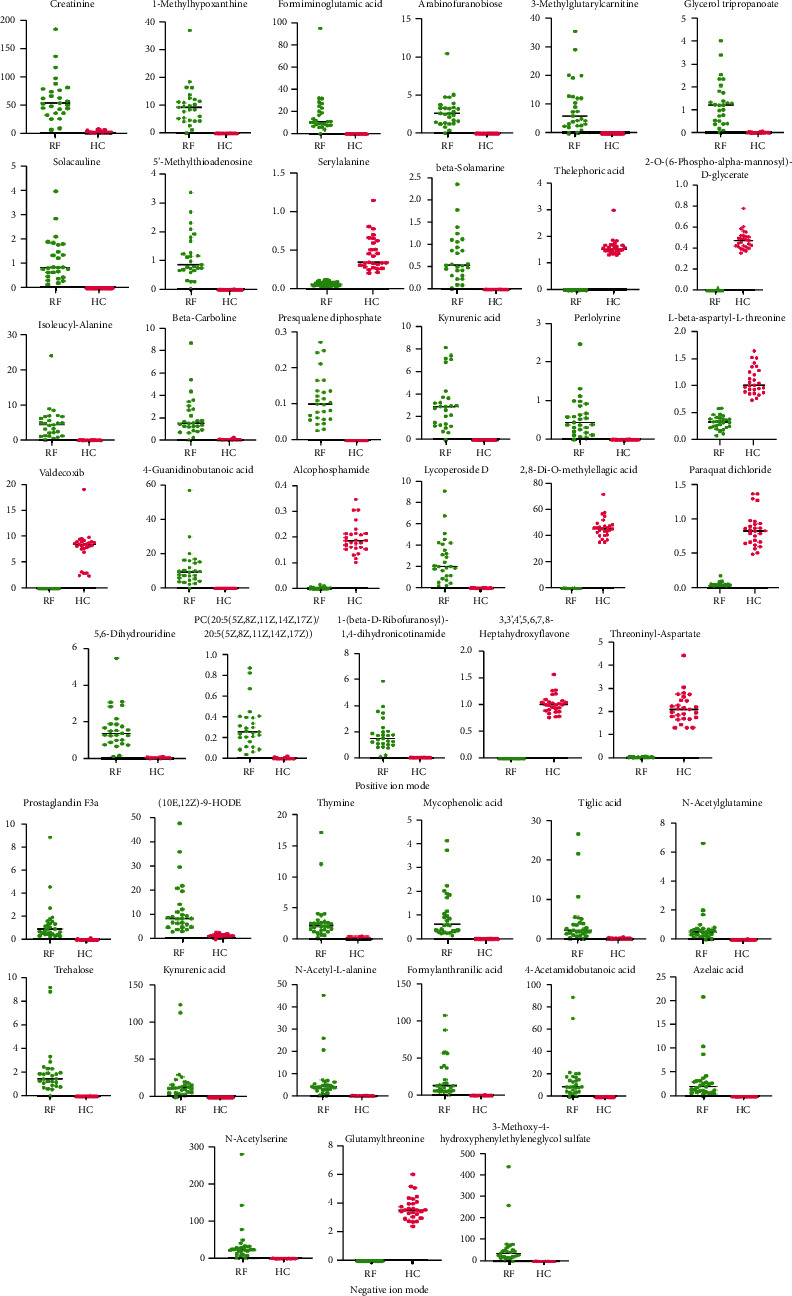
Dot maps of metabolites in positive and negative ion modes.

**Figure 5:**
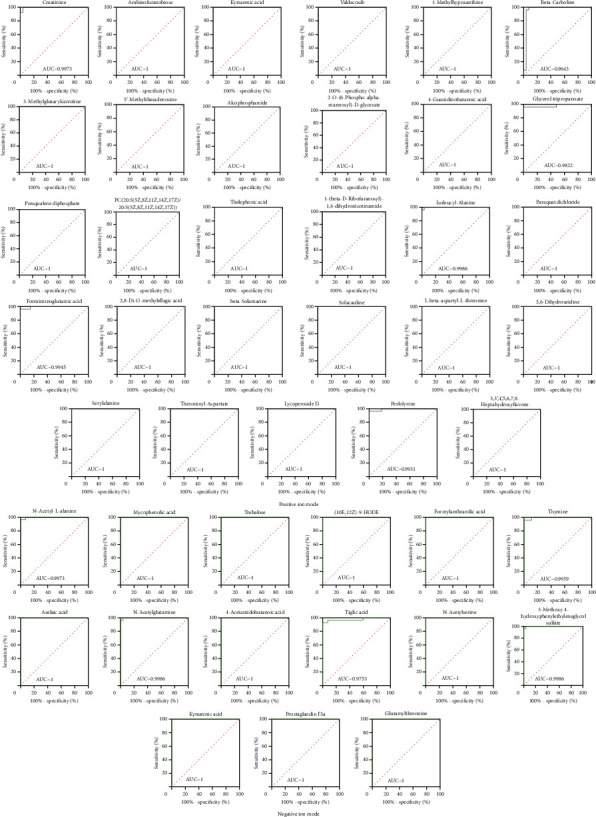
ROC curves of metabolites in positive and negative ion modes.

Based on a local database with MS/MS fragment information, a total of 141 and 119 metabolites were identified from the positive and negative ion modes of RF, respectively, as compared to the health control (Positive ion mode: Supplementary Table S2, Negative ion mode: Supplementary Table S3). Volcano plots are shown in Figure 2, and increased abundance is indicated in red, whereas decreased abundance is indicated in blue. Excluding the nonendogenous metabolites based on the HMDB database, the endogenous metabolites were remaining 118 and 104, respectively. By comparing the metabolites between positive and negative modes, 6 endogenous metabolites are shared by both, including creatinine, kynurenic acid, 4-pyridoxic acid, 4-hydroxyproline, prolylhydroxyproline, and phenylacetylglutamine, as shown in Figure 3. All the differential metabolites could be classified into 7 different categories, of which the largest number was amino acids, followed by fatty acids and alkaloids (Table 3). Based on relative quantification, we established the dot plots of all differential endogenous metabolites (Supplementary Figure S3). It was found that the response values of some metabolites which came from the RF group were similar to those from the HC group. The significant difference between these metabolites between the RF and HC groups was caused by a higher dispersion, which will influence the clinical utility of subsequent biomarkers in RF disease. Therefore, we selected metabolites for which the response values were totally different between RF and HC groups to establish the ROC curves, and the metabolites with an area under the curve (AUC) value close to 1 were screened out as potential biomarkers. In the positive and negative ion modes, we screened 29 and 15 metabolites respectively, as shown in Table 4, and only one metabolite, kynurenic acid, was shared by both. The dot diagrams and ROC curves were shown in Figures 4 and 5, respectively.

### 3.3. Hierarchical Cluster Analysis of Differential Metabolites

The differential metabolites obtained through the above strategies often exhibited functional similarities/complementarities in physics or were subjected to positive and negative regulation of the same metabolic pathways. HCA of such characteristics could help us classify metabolites with the same characteristics into one category and discover the characteristics of changes in metabolites between RF and HC groups. All the differential metabolites were clustered using HCA, and the results were displayed in a heat map in Supplementary Figure S4A (positive) and S4B (negative). Intuitively, all the samples could be clustered together based on the significantly increased and decreased metabolites of the RF group as compared with the HC group, and the number of increased metabolites was significantly more than the decreased metabolites.

### 3.4. KEGG Annotation and Metabolic Pathway Analysis of Differential Metabolites

**Figure 6:**
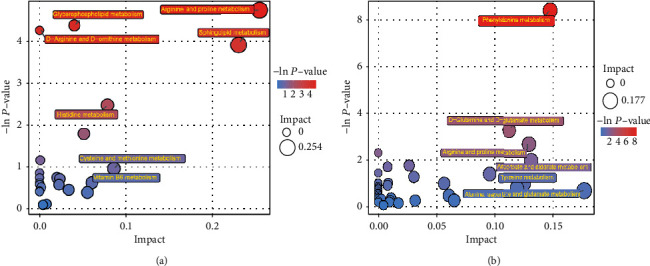
Pathway analysis of group RF vs HC. The results of metabolic pathway analysis are displayed in bubble blot. (a) presents positive ion mode, (b) presents negative ion mode.

Generally, complex metabolic reactions and regulations in objects did not go through alone, and different genes and proteins often formed complex networks of signaling pathways. Their mutual influences and regulations eventually lead to systemic changes in the metabolome. The analysis of these metabolites and regulatory pathways could provide a more comprehensive and systematic understanding of the changes in the physical process, as well as the mechanisms of the occurrence of traits or diseases. Therefore, after obtaining the matching information of the different metabolites, the metabolic pathways were analyzed based on the pathway library of *Homo sapiens* (human). Then, the pathway diagram of the differential metabolites mapped by the KEGG database is described in Supplementary Figure S5. However, as the diagram shows, the pathways involved all the different metabolites were so complicated and difficult to clarify the relationships among them, thus the enrichment and topological analysis on those pathways were conducted, as shown in Figure 6. Specifically, arginine and proline metabolism, sphingolipid metabolism, glycerophospholipid metabolism, D-arginine and D-ornithine metabolism were the main differential metabolic pathways in positive ion mode (Supplementary Figures S6a–S6d). While in negative ion mode, phenylalanine metabolism, ascorbate and aldarate metabolism, D-glutamine and D-glutamate metabolism, arginine and proline metabolism (Supplementary Figures S7a–S7d) were the main differential metabolic pathways. Arginine and proline metabolism were the common pathways in both positive and negative ion modes.

### 3.5. Random Forest Analysis of Differential Metabolites

**Figure 7:**
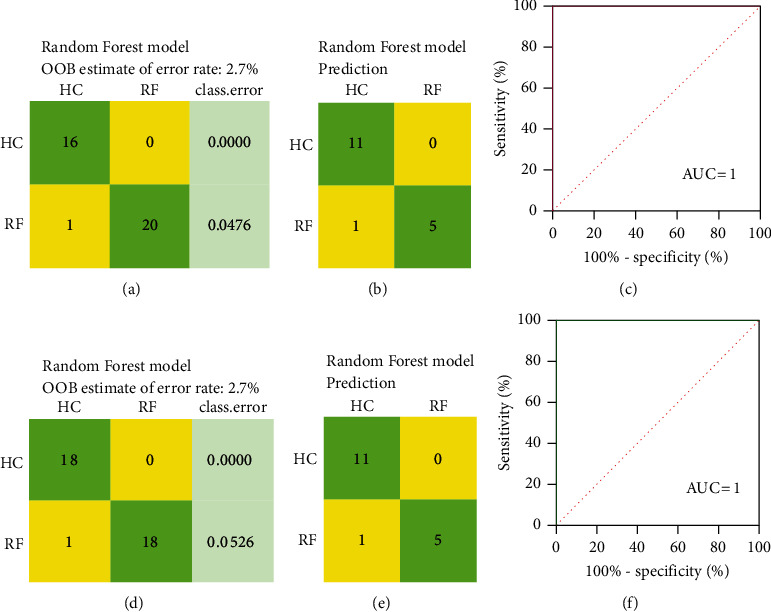
Random forest model of differential metabolites of the RF group vs HC group. (a) and (d) are the classification confusion matrix of random forest models in the training set; (b) and (e) are the classification confusion matrices of random forest models in the prediction set. (c) and (f) are the ROC curves based on the prediction probability of the random forest model. (a), (b), and (c) show the analysis of differential metabolites screened by positive ion mode. (d), (e) and (f) show the analysis of differential metabolites screened by negative ion mode.

**Figure 8:**
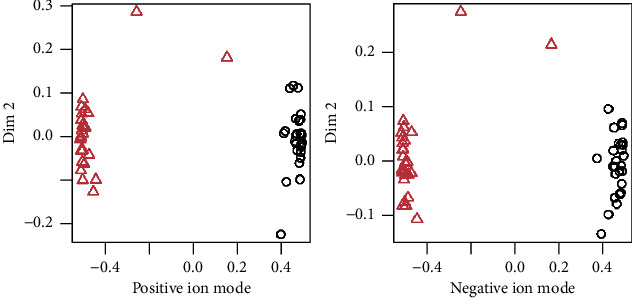
Random forest coordinate map.

To further verify the classification effect of the different metabolites between RF and HC groups, we trained the random forest model, and the training results are shown in Figure 7. The out-of-bag errors were equal to 2.7% in positive and negative ion modes, and the values of AUC were all equal to 1 in both positive and negative ion modes. Based on the coordinate map (Figure 8) of the random forest model, it could be seen that the two sets of samples were clustered separately, indicating that the established random forest models exhibited excellent discriminative efficiency.

## 4. Discussion

RF is a pathological condition that causes partial or complete loss of renal function and is stimulated by the development of various kidney diseases. In our present study, various changes in amino acid metabolites, nucleic acid metabolites, and glycometabolism metabolites were found in the serum of RF patients as compared to HC. Generally, while the amino acid are within the normal range, it has been assumed that these changes are due to low protein intake and deficiency of excretory and metabolic functions of the diseased kidneys. According to the published studies, significant changes in amino acid metabolites in ESRD patients have been observed, which implies that the changes in amino acid metabolisms were already detectable at an early stage of RF [16]. Metabolites might be varied because of differences in tubular secretion and resorption, as well as kidney catabolism and anabolism. However, even perfect adjustment for eGFR might not fully address the effect of kidney function on normal metabolite levels [10]. Therefore, this study employed untargeted metabolomics to measure and quantify the metabolites as much as possible that might be used in the diagnosis and treatment of kidney disease. In our experiments, a total of 216 differential metabolites were elucidated (the typical mass spectra of metabolites were presented in Supplementary Figure S8), and 44 metabolites showed high sensitivity and specificity which might be used as serum biomarkers for RF. From these 44 potential biomarkers, it could be seen that RF was mainly associated with 7 metabolic pathways: (1) arginine and proline metabolism; (2) sphingolipid metabolism; (3) glycerophospholipid metabolism; (4) D-arginine and D-ornithine metabolism; (5) phenylalanine metabolism; (6) ascorbate and aldarate metabolism; and (7) D-glutamine and D-glutamate metabolism. Based on the HMDB database, 4 of 7 metabolic pathways involved amino acid metabolism, and 11 of 44 potential biomarkers belonged to amino acids derivatives, including creatinine, 4-guanidinobutanoic acid, formiminoglutamic acid, serylalanine, isoleucyl-alanine, threoninyl-aspartate, L-beta-aspartyl-L-threonine, 4-acetamidobutanoic acid, N-acetyl-L-alanine, N-acetylserine, and glutamylthreonine. Meanwhile, only 3 of 11 metabolites including serylalanine, threoninyl-aspartate, and glutamylthreonine were downregulated in RF disease, while the others were all upregulated. The above metabolite changes reflected that the occurrence of RF was mainly related to the metabolic disorders of amino acid metabolism, which manifested in decreased renal function, insufficient nutrient intake, accumulation of urinary toxins, intestinal flora disturbance, endocrine disorder, etc.

Rhee EP et al raised the hypothesis that arginine might be developed as a marker of renal metabolic function whose plasma level could provide insight on renal prognosis [21]. In our study, two metabolic pathways involving arginine were found, but arginine was not screened as a differential metabolite, which might be related to our small sample size. In addition, it has been reported that the kidney is exquisitely sensitive to sphingolipid metabolism. The dysfunctional sphingolipid metabolism might cause complications of renal disease [22]. From the sphingolipid metabolism graph, we also see that phytosphingosine, sphingaine, and sphingomyeline have different degrees of aggregation. These metabolites were related to the regulation of renal function during RF [23]. In our study, the level of PC (20 : 5 (5Z, 8Z, 11Z, 17Z)/(20 : 5 (5Z, 8Z, 11Z, 17Z)) was obviously increased in clinical patient with RF, suggesting that RF might be accompanied with glycerophospholipid metabolism disorder. Moreover, among these 44 potential markers, 3 metabolites (creatinine, thymine, and kynurenic acid) have been recorded in the European uremic solute database. Studies have demonstrated that creatinine is an important indicator for clinical diagnosis of RF. The level of creatinine was significantly increased when RF occurred, which was in accordance with the result of our study. Kynurenic acid, a product of tryptophan metabolism, has also been recognized as a protein-bound uremic toxin. The accumulation of kynurenic acid could be found in the early stage of CKD, thus the level of kynurenic acid might be directly proportional to the progression of kidney disease. Accumulating evidence has found that the blood level of kynurenic acid was increased in patients with CKD, which was consistent with our result [24]. In addition, previous studies [19, 25] have shown that the level of 5′-methylthioadenosine was significantly increased in clinical patients with RF, which was also in agreement with our findings.

## 5. Conclusions

Briefly, by employing a Q-Exactive HFX Orbitrap LC-MS/MS based untargeted metabolomics, using PCA, PLS-DA, HCA, KEGG annotation, metabolic pathway, and random forest analysis, and by establishing ROC and dot plots, we discovered 44 metabolites in RF with high sensitivity and specificity. The metabolic profile we elucidated might provide preliminary comprehensive insight into the molecular basis of RF. More importantly, these metabolites might be used as serum biomarkers in the diagnosis and treatment of RF, as well as open a new perspective for subsequent research.

In addition, in our present study, untargeted metabolomics seemed to be a useful approach to discover the potential metabolic indicators of RF disease. However, our study also exposed some limitations that should be marked. The first one considered the small sample size of the studied groups. On the other hand, the observed serum metabolomic profiles might also be influenced by some exogenous factors like diet, applied pharmacotherapy, or comorbidities present in both RF and the health control groups. Therefore, further prospective evaluation with an expanding clinical sample quantity is needed to validate the clinical value and accuracy of the potential metabolic biomarkers scrutinized in the present study in RF disease diagnosis and treatment.

## Figures and Tables

**Table 1:** Sample information of the RF and control group.

Sample information	RF	HC	All
Sample size	27	27	54

Gender			
Female	5	13	18
Male	22	14	36

Age			
<20	0	0	0
20–29	2	2	4
30–39	7	7	14
40–49	8	8	16
50–59	6	7	13
≥60	4	3	7

**Table 2:** Sample characteristics of the RF.

	*Characteristics*	*RF*

	BMI (kg/m^2^)	23.07 ± 3.72

Liver function	ALT (U/L)	12.14 ± 8.62
	AST (U/L)	13.33 ± 6.26
	AST/ALT	1.47 ± 0.84

Renal function	Ur	20.33 ± 5.23
	Cr (*μ*mol/L)	937.10 ± 250.72
	Scr (mg/dl)	10.60 ± 2.84
	eGFR (mL/min/1.73 m^2^	5.35 ± 1.81
	UA (*μ*mol/L)	442.58 ± 111.41

Blood glucose	GLU (mmol/L)	7.57 ± 5.39

Blood lipids	TG (mmol/L)	1.98 ± 1.62
	TC (mmol/L)	3.15 ± 0.63
	HDL-C (mmol/L)	1.01 ± 0.36
	LDL-C (mmol/L)	2.38 ± 0.589

*Past medical history*	*Treat/Not treat*	*RF*
Coronary heart disease	Treated	13 (48.14%)
Diabetes	Treated	5 (18.52%)
Renal hypertension	Treated	11 (40.74)
No disease		3 (11.11%)

**Table 3:** Categories of potential renal failure metabolites.

	Amino acid	Fatty acids	Glycerophospholipids	Alkaloids	Nucleosides	Carbohydrate	Other	Total
POS	23	10	9	9	3	1	63	118
NEG	22	17	0	2	5	8	50	104

**Table 4:** Potential renal failure metabolites.

No	Metabolites	Level in patients	AUC
1	Creatinine^*∗*^	↑	0.997
2	1-methylhypoxanthine	↑	1
3	Beta-carboline	↑	0.964
4	Arabinofuranobiose	↑	1
5	Valdecoxib	↓	1
6	Glycerol tripropanoate	↑	0.982
7	4-Guanidinobutanoic acid	↑	1
8	Kynurenic acid^*∗*^	↑	1
9	Alcophosphamide	↓	1
10	1-(Beta-D-ribofuranosyl)-1, 4-dihdronicotinamide	↑	1
11	Thelephoric acid	↓	1
12	5′-Methylthioadenosine^*∗*^	↑	1
13	3-Methylglutarylcarnitine	↑	1
14	Formiminoglutamic acid	↑	0.994
15	Solacauline	↑	1
16	PC (20 : 5 (5Z, 8Z, 11Z, 17Z)/(20 : 5 (5Z, 8Z, 11Z, 17Z))	↑	1
17	Serylalanine	↓	1
18	5, 6-Dihydrouridine	↑	1
19	L-Beta-aspartyl-L-threonine	↓	1
20	Isoleucyl-alanine	↑	0.999
21	Beta-solamarine	↑	1
22	Presqualene diphosphate	↑	1
	Lycoperoside D	↑	1
24	2-O-(6-Phospho-alpha-mannosyl)-D-glycerate	↓	1
25	2, 8-Di-O-methylellagic acid	↓	1
26	Perlolyrine	↑	0.993
27	3, 3′, 4′, 5, 6, 7, 8-Heptahydroxyflavone	↓	1
28	Threoninyl-aspartate	↓	1
29	Paraquat dichloride	↓	1
30	Azelaic acid	↑	1
31	(10E, 12Z)-9-HODE	↑	1
32	N-acetylglutamine	↑	1
33	4-acetamidobutanoic acid	↑	1
34	N-acetyl-L-alanine	↑	0.997
35	Mycophenolic acid	↑	1
36	Formylanthranilic acid	↑	1
37	Trehalose	↑	1
38	Prostaglandin F3a	↑	1
39	Thymine	↑	0.996
40	Kynurenic acid	↑	1
41	Tiglic acid	↑	0.975
42	N-acetylserine	↑	1
43	Glutamyltheronine	↓	1
44	3-methoxy-4-hydroxyphenylethyleneglycol sulfate	↑	0.999

^*∗*^ presents the metabolites identified in kidney disease had been reported.

## Data Availability

All data, models, and code generated or used during the study are available in this study.

## Supplementary Materials

Supplementary Materials Supplementary Figure S1: Quality control diagram. A and B are TIC diagrams of QC samples; C and D are EIC diagrams of internal standard in QC sample; E and F are PCA analysis of QC samples. Supplementary Figure S2: Correlation analysis heat map in positive and negative modes, respectively. Supplementary Figure S3: Dot map of all the different endogenous metabolites. Supplementary Figure S4: Heatmap of hierarchical clustering analysis of group RF vs HC. Supplementary Figure S5: Metabolic pathways with red/blue dots representing the differentially expressed compounds. Red means up regulation, blue means down regulation. Supplementary Figure S6: a KEGG metabolic pathway, Arginine and Proline metabolism. Supplementary Figure S6: b KEGG metabolic pathway, Sphingolipid metabolism. Supplementary Figure S6: c KEGG metabolic pathway, Glycerophospholipid metabolism. Supplementary Figure S6: d KEGG metabolic pathway, D-Arginine and D-ornithine metabolism. Supplementary Figure S7: a KEGG metabolic pathway, Phenylalanine metabolism. Supplementary Figure S7: b KEGG metabolic pathway, Ascorbate and aldarate metabolism. Supplementary Figure S7: c KEGG metabolic pathway, D-Glutamine and D-glutamate metabolism. Supplementary Figure S7: d KEGG metabolic pathway, Arginine and proline metabolism. Supplementary Figure S8: The typical mass spectra of metabolites. Supplementary Table S1: The clinical characteristics of patients. Supplementary Table S2: POS-Differentially Expressed Metabolites. Supplementary Table S3: NEG-Differentially Expressed Metabolites.

## References

[1] Rhee E. P., Souza A., Farrell L. (2010). Metabolite profiling identifies markers of uremia. *Journal of the American Society of Nephrology*.

[2] Wang X., Yang S., Li S. (2020). Aberrant gut microbiota alters host metabolome and impacts renal failure in humans and rodents. *Gut*.

[3] Gagnebin Y., Pezzatti J., Lescuyer P., Boccard J., Ponte B., Rudaz S. (2019). Toward a better understanding of chronic kidney disease with complementary chromatographic methods hyphenated with mass spectrometry for improved polar metabolome coverage. *Journal of Chromatography B*.

[4] Hocher B., Adamski J. (2017). Metabolomics for clinical use and research in chronic kidney disease. *Nature Reviews Nephrology*.

[5] Chen D. Q., Cao G., Chen H. (2019). Identification of serum metabolites associating with chronic kidney disease progression and anti-fibrotic effect of 5-methoxytryptophan. *Nature Communications*.

[6] Cui L., Lu H., Lee Y. H. (2018). Challenges and emergent solutions for LC-MS/MS based untargeted metabolomics in diseases. *Mass Spectrometry Reviews*.

[7] Nkuipou-Kenfack E., Duranton F., Gayrard N. (2014). Assessment of metabolomic and proteomic biomarkers in detection and prognosis of progression of renal function in chronic kidney disease. *PLoS One*.

[8] Kalim S., Rhee E. P. (2017). An overview of renal metabolomics. *Kidney International*.

[9] Kanda H., Hirasaki Y., Iida T. (2017). Perioperative management of patients with end-stage renal disease. *Journal of Cardiothoracic and Vascular Anesthesia*.

[10] Grams M. E., Shafi T., Rhee E. P. (2018). Metabolomics research in chronic kidney disease. *Journal of the American Society of Nephrology*.

[11] Rahman M., Islam R., Rabbi F. (2022). Bioactive compounds and diabetes mellitus: prospects and future challenges. *Current Pharmaceutical Design*.

[12] Rahman M. M., Islam M. R., Shohag S. (2022). The multifunctional role of herbal products in the management of diabetes and obesity: a comprehensive review. *Molecules*.

[13] Rahman M. M., Rahaman M. S., Islam M. R. (2021). Role of phenolic compounds in human disease: current knowledge and future prospects. *Molecules*.

[14] Rahman M. M., Dhar P. S., Sumaia N. (2022). Exploring the plant-derived bioactive substances as antidiabetic agent: an extensive review. *Biomedicine & Pharmacotherapy*.

[15] Rahman M. M., Bibi S., Rahaman M. S. (2022). Natural therapeutics and nutraceuticals for lung diseases: traditional significance, phytochemistry, and pharmacology. *Biomedicine & Pharmacotherapy*.

[16] Hayashi K., Sasamura H., Hishiki T., Suematsu M., Soga T., Itoh H. (2011). Use of serum and urine metabolome analysis for the detection of metabolic changes in patients with stage 1-2 chronic kidney disease. *Nephro-Urology Monthly*.

[17] Kwan B., Fuhrer T., Zhang J. (2020). Metabolomic markers of kidney function decline in patients with diabetes: evidence from the chronic renal insufficiency cohort (CRIC) study. *American Journal of Kidney Diseases*.

[18] Rhee E. P. (2015). Metabolomics and renal disease. *Current Opinion in Nephrology and Hypertension*.

[19] Gagnebin Y., Jaques D. A., Rudaz S., de Seigneux S., Boccard J., Ponte B. (2020). Exploring blood alterations in chronic kidney disease and haemodialysis using metabolomics. *Scientific Reports*.

[20] Velenosi T. J., Thomson B. K. A., Tonial N. C. (2019). Untargeted metabolomics reveals N, N, N-trimethyl-L-alanyl-L-proline betaine (TMAP) as a novel biomarker of kidney function. *Scientific Reports*.

[21] Rhee E. P., Clish C. B., Wenger J. (2016). Metabolomics of chronic kidney disease progression: a case-control analysis in the chronic renal insufficiency cohort study. *American Journal of Nephrology*.

[22] Bhat O. M., Yuan X., Li G., Lee R., Li P. L. (2018). Sphingolipids and redox signaling in renal regulation and chronic kidney diseases. *Antioxidants and Redox Signaling*.

[23] Mitrofanova A., Drexler Y., Merscher S., Fornoni A. (2020). Role of sphingolipid signaling in glomerular diseases: focus on DKD and FSGS. *J Cell Signal*.

[24] Pawlak K., Domaniewski T., Mysliwiec M., Pawlak D. (2009). Kynurenines and oxidative status are independently associated with thrombomodulin and von Willebrand factor levels in patients with end-stage renal disease. *Thrombosis Research*.

[25] Zhang Z. H., Vaziri N. D., Wei F., Cheng X. L., Bai X., Zhao Y. Y. (2016). An integrated lipidomics and metabolomics reveal nephroprotective effect and biochemical mechanism of Rheum officinale in chronic renal failure. *Scientific Reports*.

