# Supplementary material for: Serum Biomarkers for Chronic Renal Failure Screening and Mechanistic Understanding: A Global LC-MS-Based Metabolomics Research
Source: Evid Based Complement Alternat Med. 2022 Jul 30;2022:7450977. doi: 10.1155/2022/7450977 (PMC9356786; doi:10.1155/2022/7450977)
Supplement: Supplementary Materials — Supplementary Figure S1: Quality control diagram. A and B are TIC diagrams of QC samples; C and D are EIC diagrams of internal standard in QC sample; E and F are PCA analysis of QC samples. Supplementary Figure S2: Correlation analysis heat map in positive and negative modes, respectively. Supplementary Figure S3: Dot map of all the different endogenous metabolites. Supplementary Figure S4: Heatmap of hierarchical clustering analysis of group RF vs HC. Supplementary Figure S5: Metabolic pathways with red/blue dots representing the differentially expressed compounds. Red means up regulation, blue means down regulation. Supplementary Figure S6: a KEGG metabolic pathway, Arginine and Proline metabolism. Supplementary Figure S6: b KEGG metabolic pathway, Sphingolipid metabolism. Supplementary Figure S6: c KEGG metabolic pathway, Glycerophospholipid metabolism. Supplementary Figure S6: d KEGG metabolic pathway, D-Arginine and D-ornithine metabolism. Supplementary Figure S7: a KEGG metabolic pathway, Phenylalanine metabolism. Supplementary Figure S7: b KEGG metabolic pathway, Ascorbate and aldarate metabolism. Supplementary Figure S7: c KEGG metabolic pathway, D-Glutamine and D-glutamate metabolism. Supplementary Figure S7: d KEGG metabolic pathway, Arginine and proline metabolism. Supplementary Figure S8: The typical mass spectra of metabolites. Supplementary Table S1: The clinical characteristics of patients. Supplementary Table S2: POS-Differentially Expressed Metabolites. Supplementary Table S3: NEG-Differentially Expressed Metabolites. [file 7450977.f1.zip › Supplementary Figure S3.pdf]

Positive ion mode

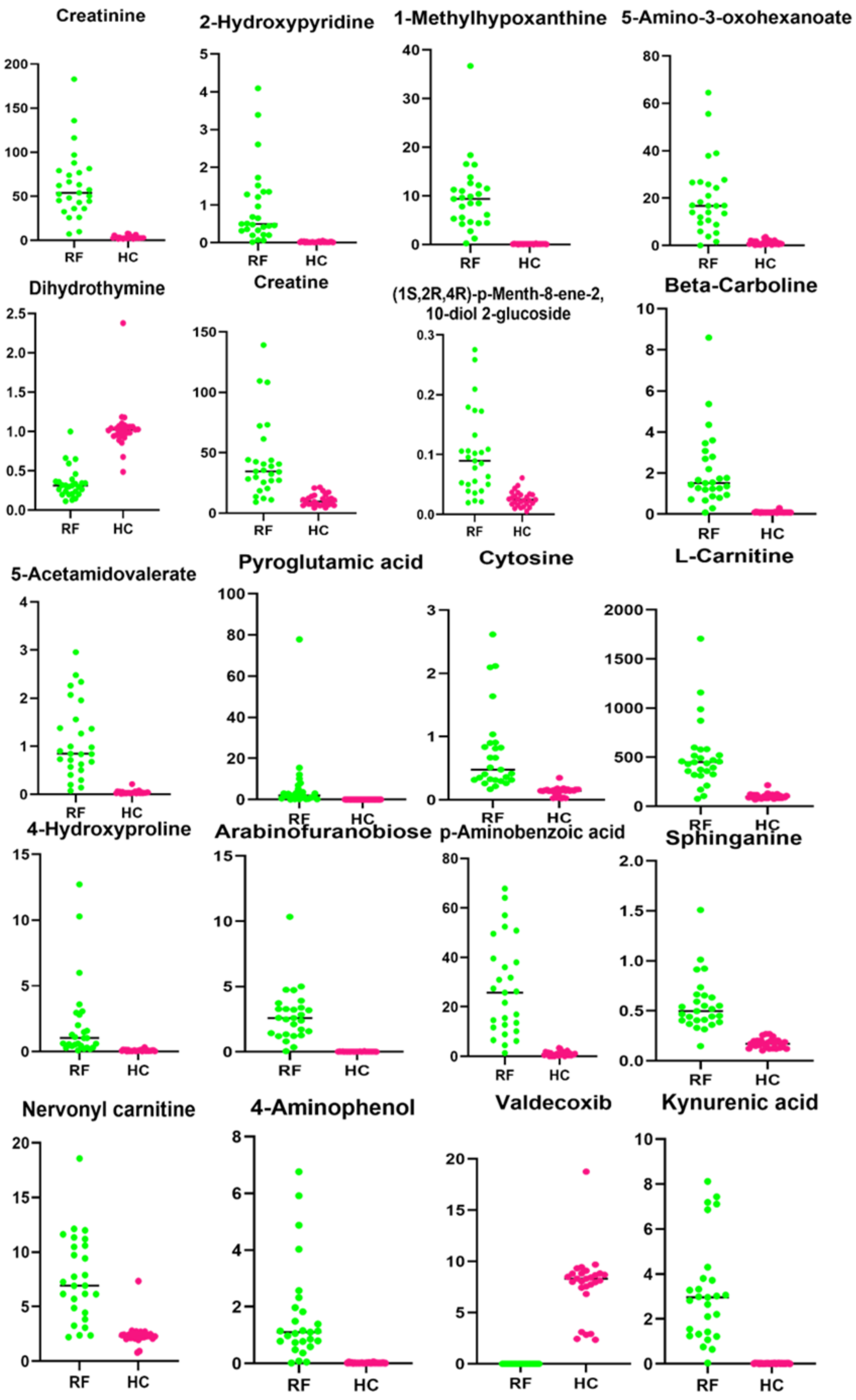

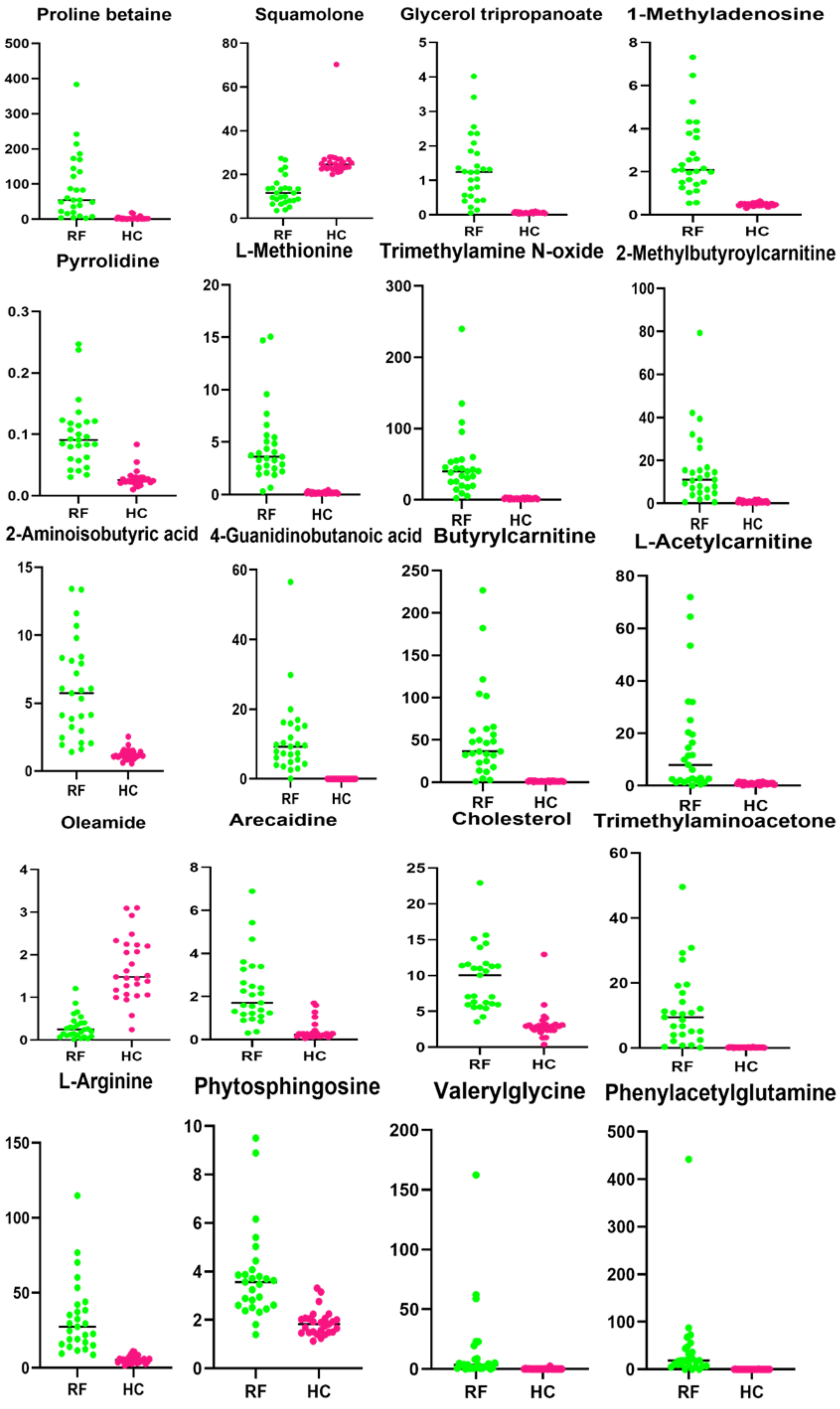
Proline betaine
Squamolone
Glycerol tripropanoate
1-Methyladenosine
RF
HC
Pyrrolidine
L-Methionine
Trimethylamine N-oxide
2-Methylbutyroylcarnitine
2-Aminoisobutyric acid
4-Guanidinobutanoic acid
Butyrylcarnitine
L-Acetylcarnitine
Oleamide
Arecaidine
Cholesterol
Trimethylaminoacetone
L-Arginine
Phytosphingosine
Valerylglycine
Phenylacetylglutamine

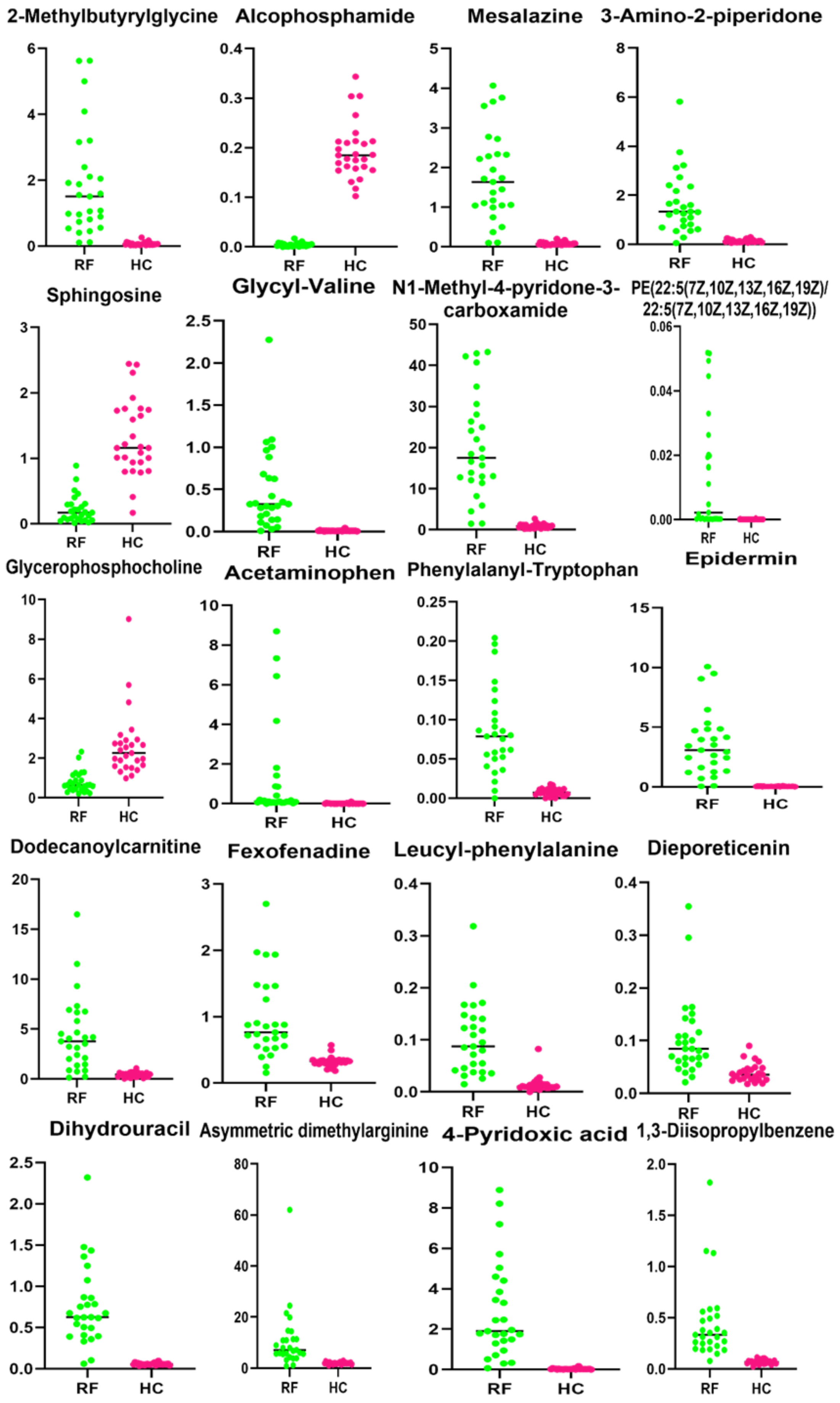
2-Methylbutyrylglycine
Alcophosphamide
Mesalazine
3-Amino-2-piperidone
Sphingosine
Glycyl-Valine
N1-Methyl-4-pyridone-3-carboxamide
PE(22:5(7Z,10Z,13Z,16Z,19Z)/22:5(7Z,10Z,13Z,16Z,19Z))
Glycerophosphocholine
Acetaminophen
Phenylalanyl-Tryptophan
Epidermin
Dodecanoylcarnitine
Fexofenadine
Leucyl-phenylalanine
Dieporeticenin
Dihydrouracil
Asymmetric dimethylarginine
4-Pyridoxic acid
1,3-Diisopropylbenzene
RF
HC

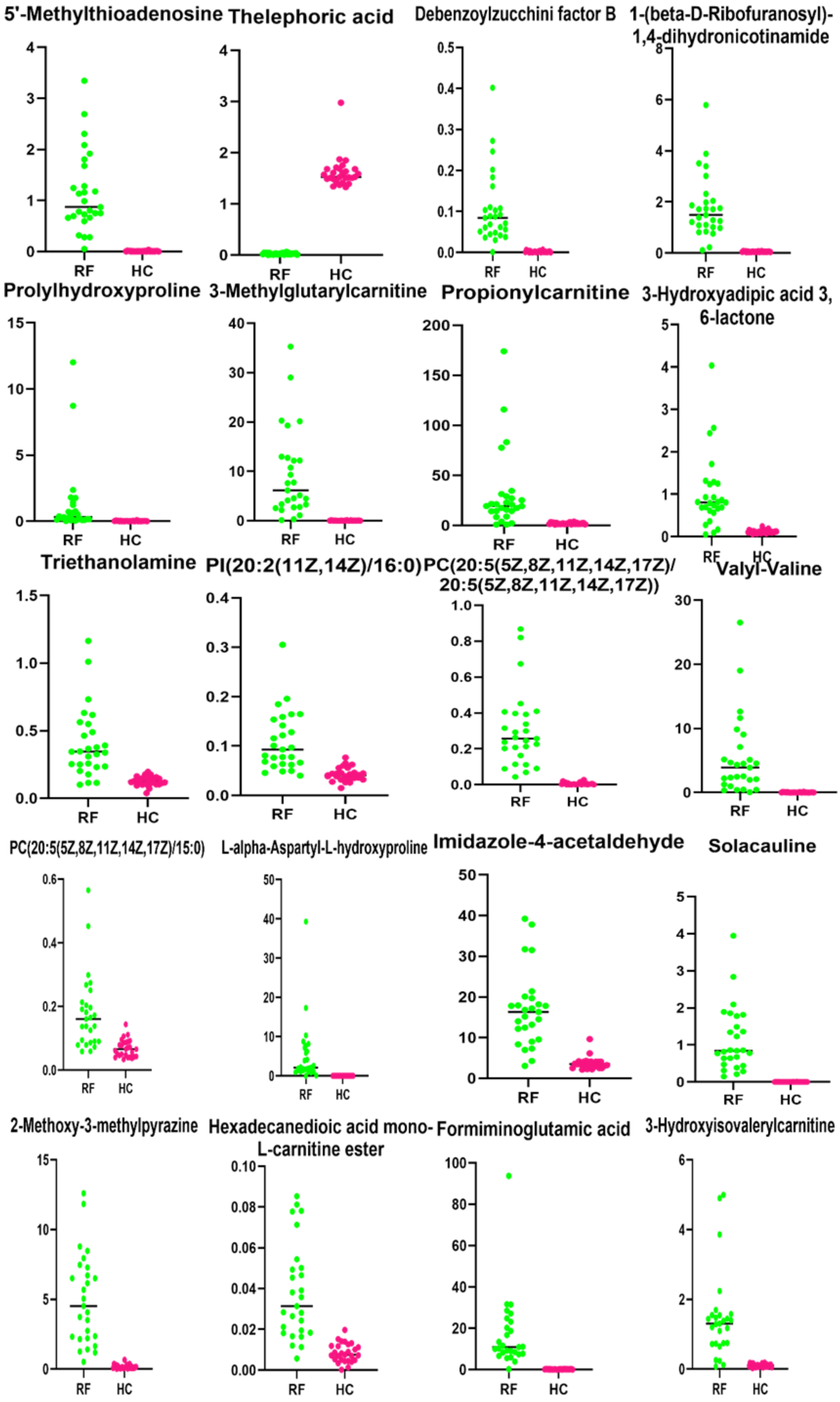
5'-Methylthioadenosine
Thelephoric acid
Debenzoylzucchini factor B
1-(beta-D-Ribofuranosyl)-1,4-dihydronicotinamide
Prolylhydroxyproline
3-Methylglutarylcarnitine
Propionylcarnitine
3-Hydroxyadipic acid 3,6-lactone
Triethanolamine
PI(20:2(11Z,14Z)/16:0)
PC(20:5(5Z,8Z,11Z,14Z,17Z)/20:5(5Z,8Z,11Z,14Z,17Z))
Valyl-Valine
PC(20:5(5Z,8Z,11Z,14Z,17Z)/15:0)
L-alpha-Aspartyl-L-hydroxyproline
Imidazole-4-acetaldehyde
Solacauline
2-Methoxy-3-methylpyrazine
Hexadecanedioic acid mono-L-carnitine ester
Formiminoglutamic acid
3-Hydroxyisovalerylcarnitine
RF
HC

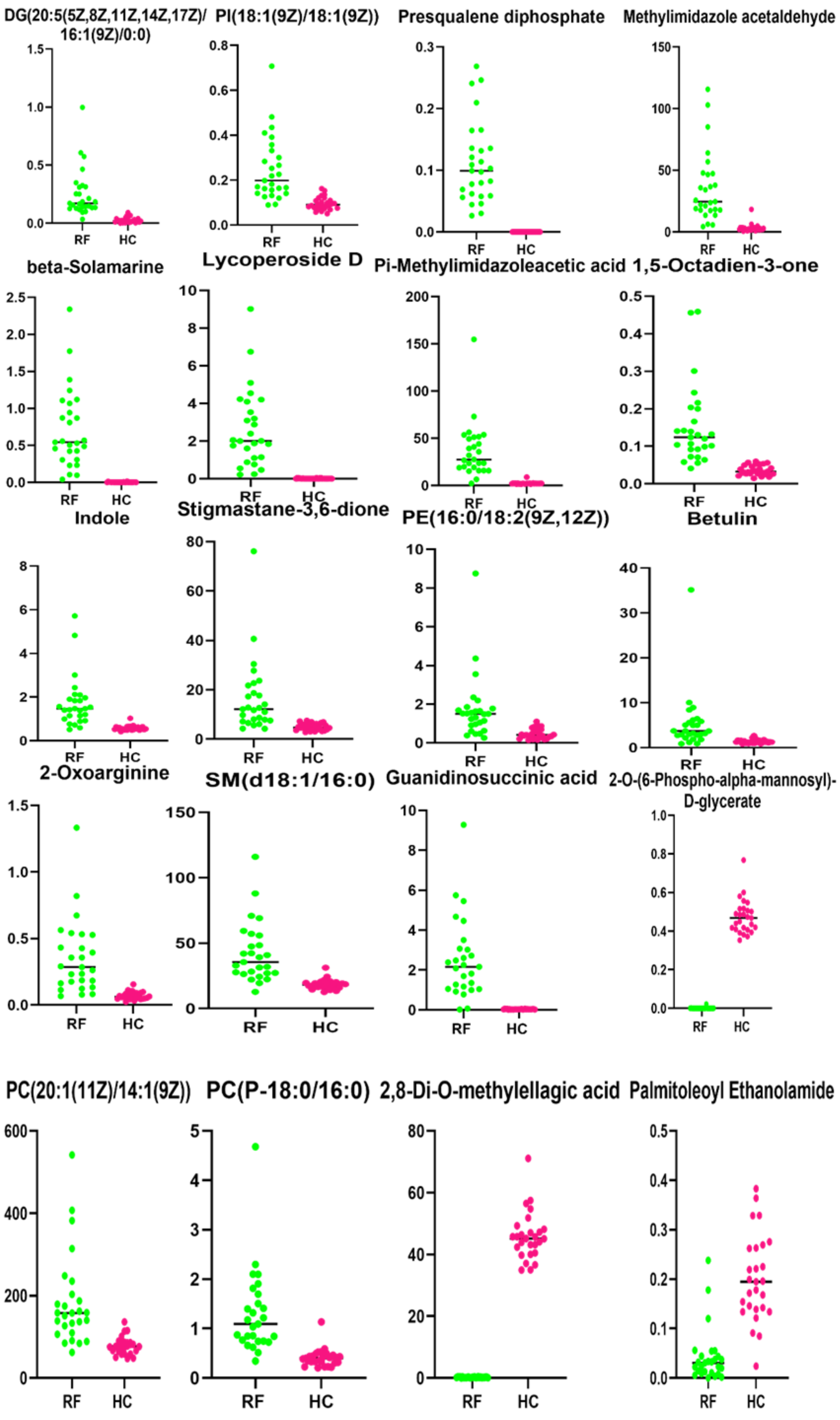
DG(20:5(5Z,8Z,11Z,14Z,17Z)/16:1(9Z)/0:0)
PI(18:1(9Z)/18:1(9Z))
Presqualene diphosphate
Methylimidazole acetaldehyde
beta-Solamarine
Lycoperoside D
Pi-Methylimidazoleacetic acid
1,5-Octadien-3-one
Indole
Stigmastane-3,6-dione
PE(16:0/18:2(9Z,12Z))
Betulin
2-Oxoarginine
SM(d18:1/16:0)
Guanidinosuccinic acid
2-O-(6-Phospho-alpha-mannosyl)-D-glycerate
PC(20:1(11Z)/14:1(9Z))
PC(P-18:0/16:0)
2,8-Di-O-methylellagic acid
Palmitoleoyl Ethanolamide
RF
HC

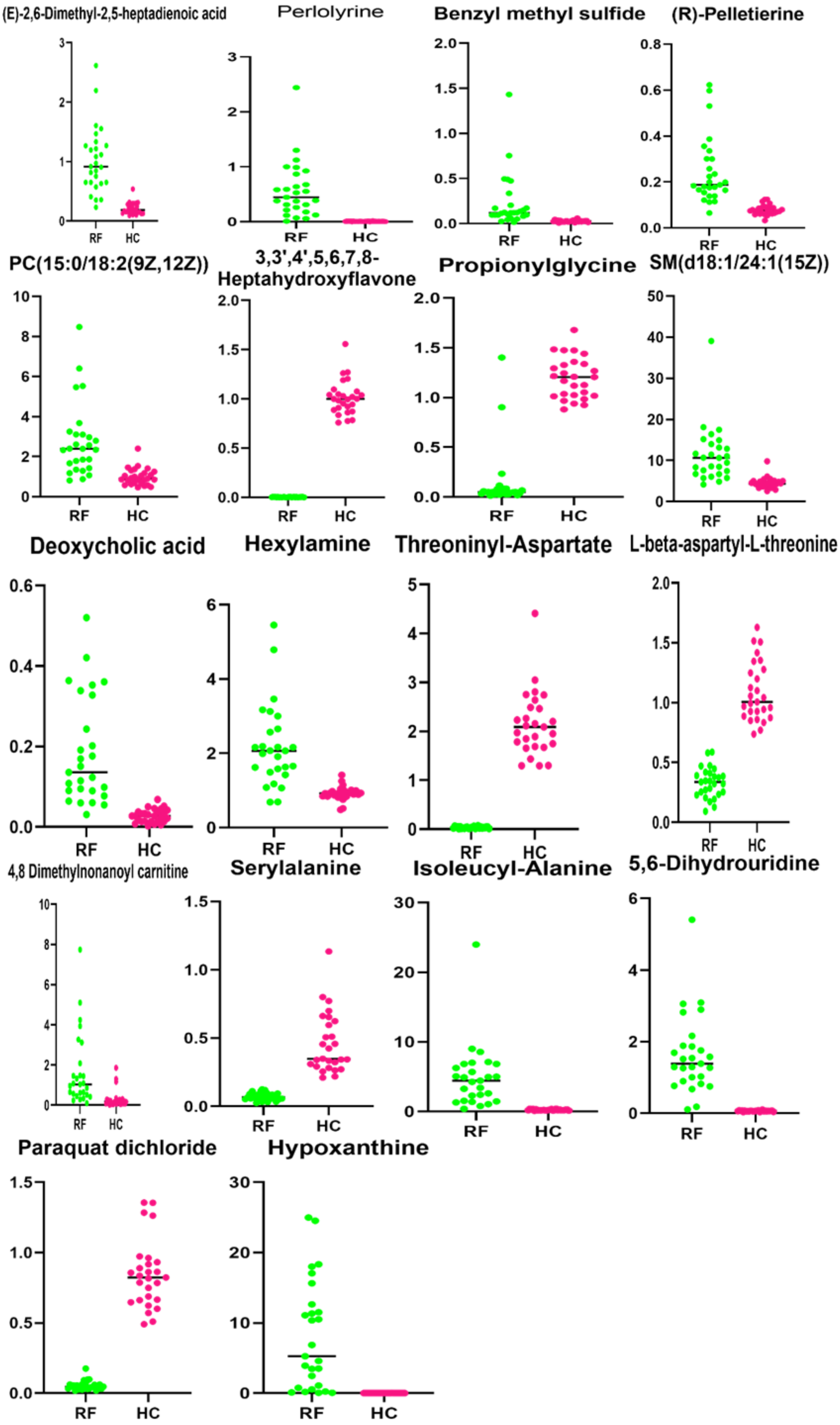
(E)-2,6-Dimethyl-2,5-heptadienoic acid
Perlolyrine
Benzyl methyl sulfide
(R)-Pelletierine
PC(15:0/18:2(9Z,12Z))
3,3',4',5,6,7,8-Heptahydroxyflavone
Propionylglycine
SM(d18:1/24:1(15Z))
Deoxycholic acid
Hexylamine
Threoninyl-Aspartate
L-beta-aspartyl-L-threonine
4,8 Dimethylnonanoyl carnitine
Serylalanine
Isoleucyl-Alanine
5,6-Dihydrouridine
Paraquat dichloride
Hypoxanthine
RF
HC

Negative ion mode

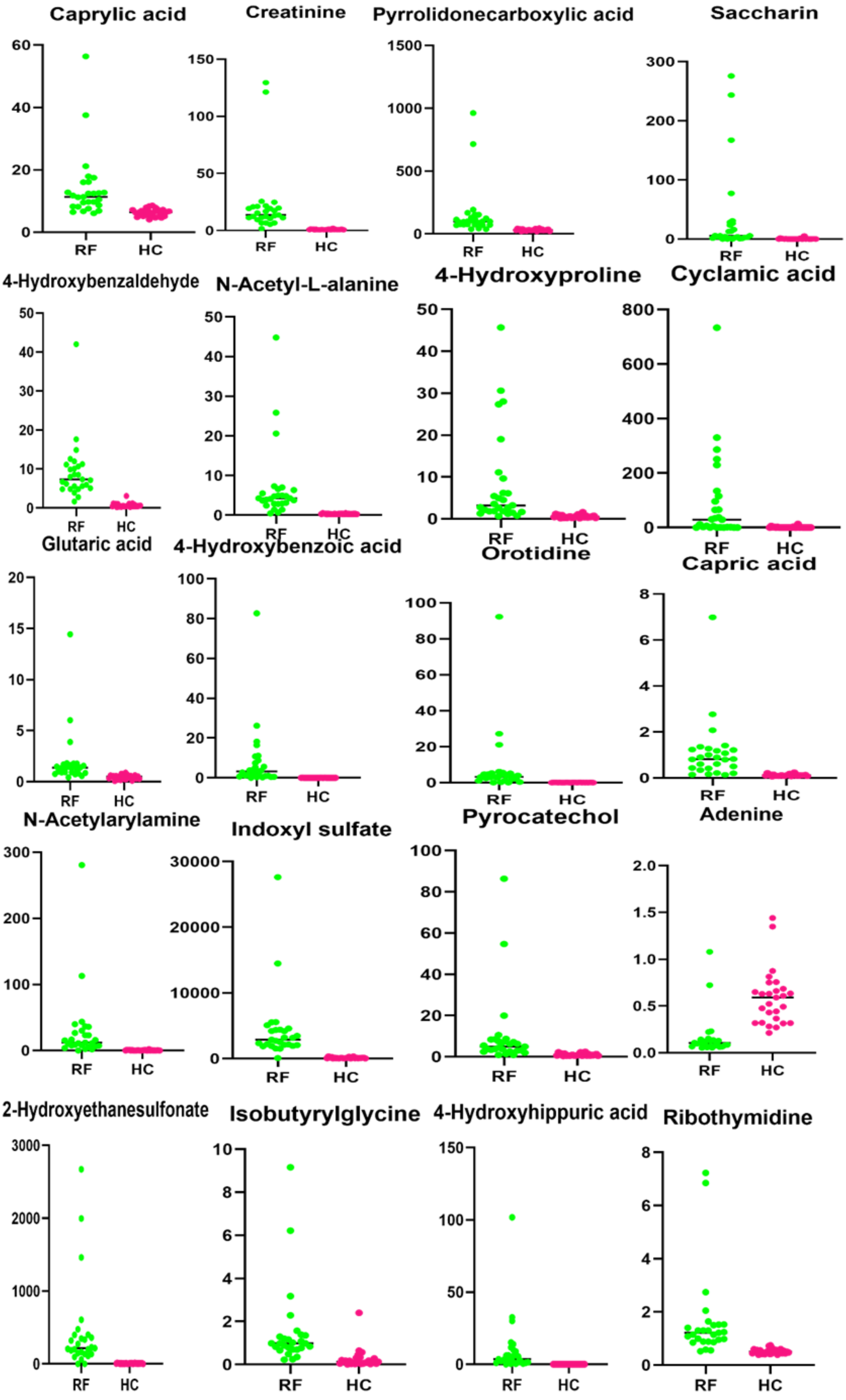

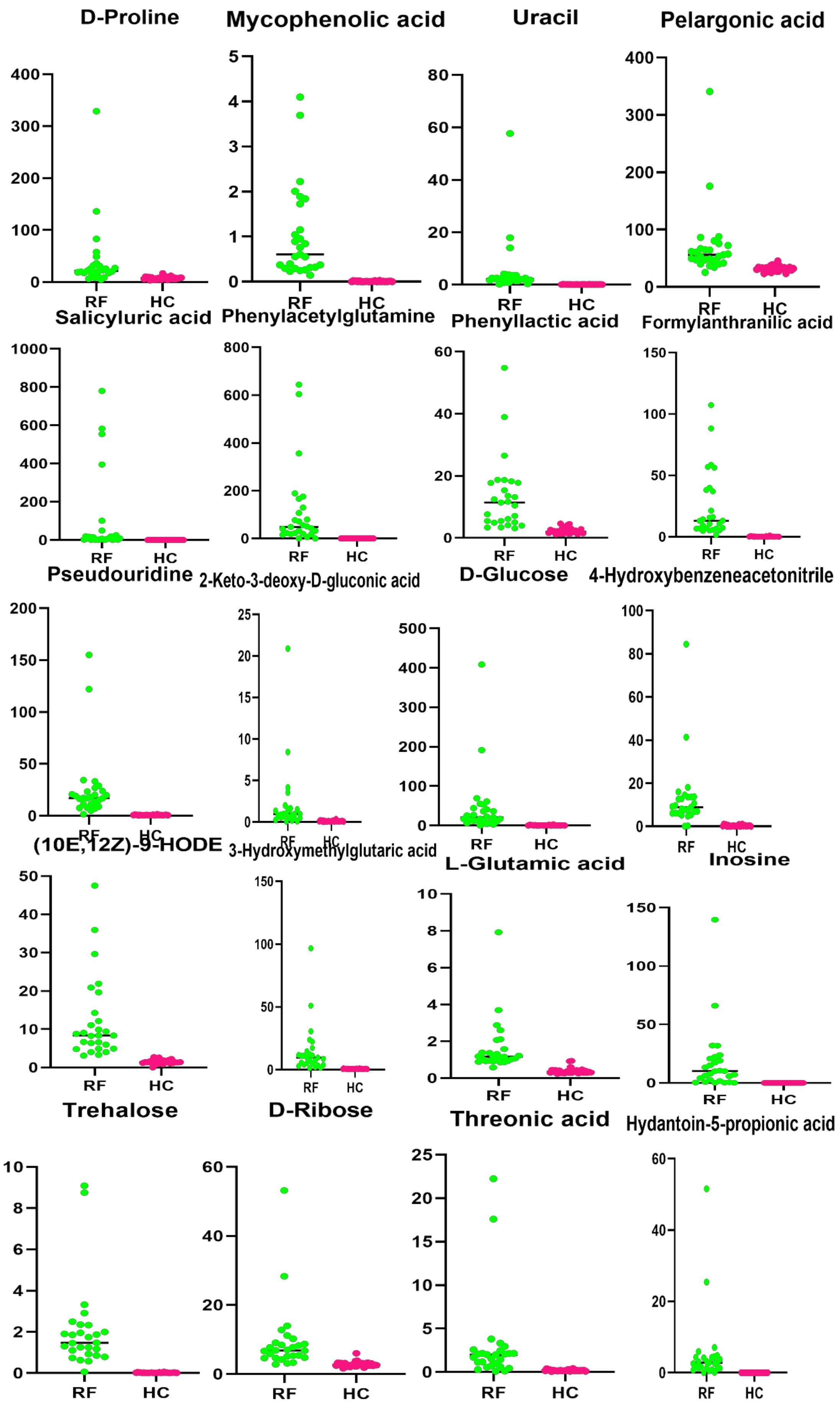
D-Proline
Mycophenolic acid
Uracil
Pelargonic acid
Salicyluric acid
Phenylacetylglutamine
Phenyllactic acid
Formylanthranilic acid
Pseudouridine
2-Keto-3-deoxy-D-gluconic acid
D-Glucose
4-Hydroxybenzeneacetonitrile
(10E,12Z)-9-HODE
3-Hydroxymethylglutaric acid
L-Glutamic acid
Inosine
Trehalose
D-Ribose
Threonic acid
Hydantoin-5-propionic acid
RF
HC

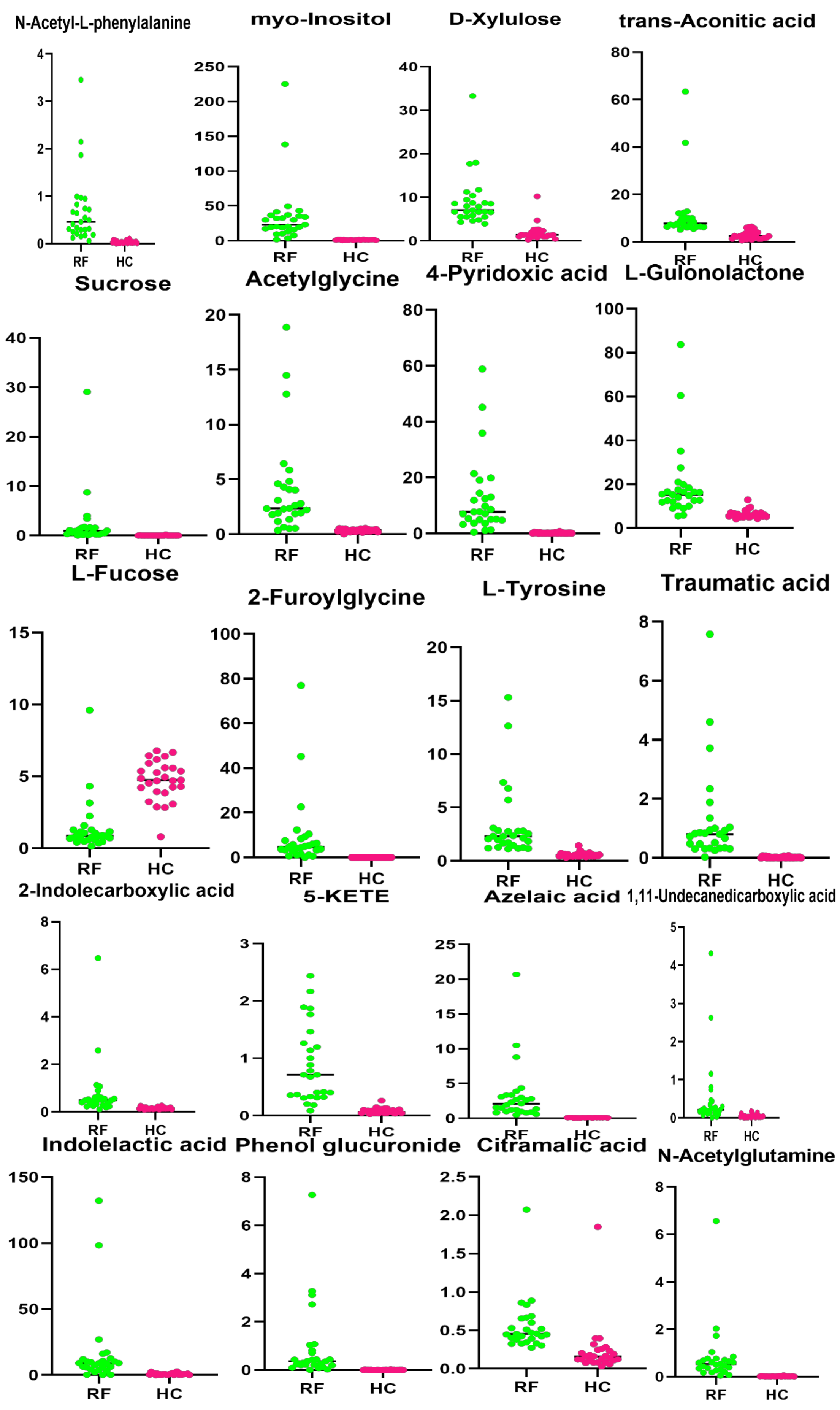
N-Acetyl-L-phenylalanine
myo-Inositol
D-Xylulose
trans-Aconitic acid
Sucrose
Acetylglycine
4-Pyridoxic acid
L-Gulonolactone
L-Fucose
2-Furoylglycine
L-Tyrosine
Traumatic acid
2-Indolecarboxylic acid
5-KETE
Azelaic acid
1,11-Undecanedicarboxylic acid
Indolelactic acid
Phenol glucuronide
Citramalic acid
N-Acetylglutamine
RF
HC

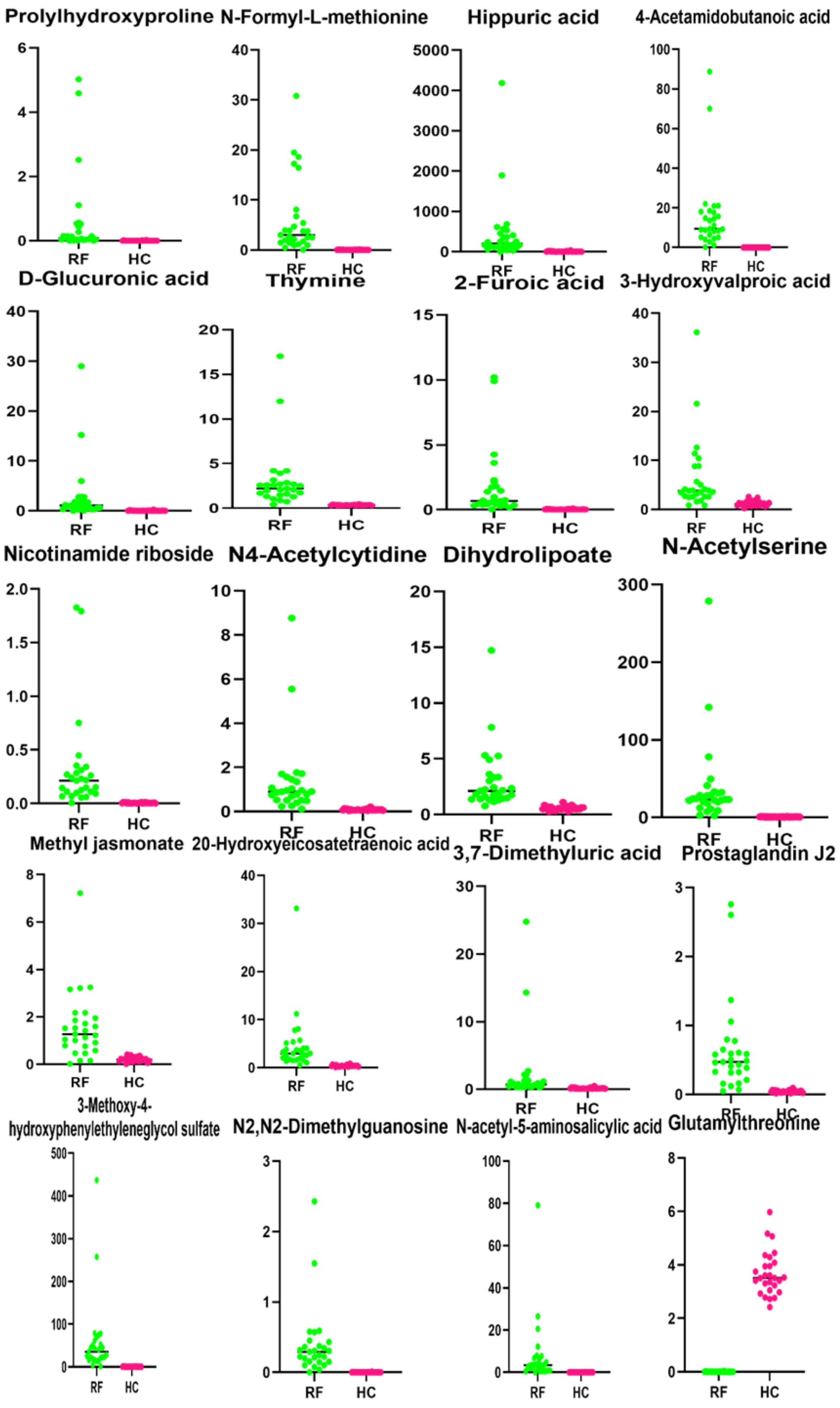
Prolylhydroxyproline
N-Formyl-L-methionine
Hippuric acid
4-Acetamidobutanoic acid
D-Glucuronic acid
Thymine
2-Furoic acid
3-Hydroxyvalproic acid
Nicotinamide riboside
N4-Acetylcytidine
Dihydrolipoate
N-Acetylserine
Methyl jasmonate
20-Hydroxyeicosatetraenoic acid
3,7-Dimethyluric acid
Prostaglandin J2
3-Methoxy-4-hydroxyphenylethyleneglycol sulfate
N2,N2-Dimethylguanosine
N-acetyl-5-aminosalicylic acid
Glutamylthreonine
RF
HC

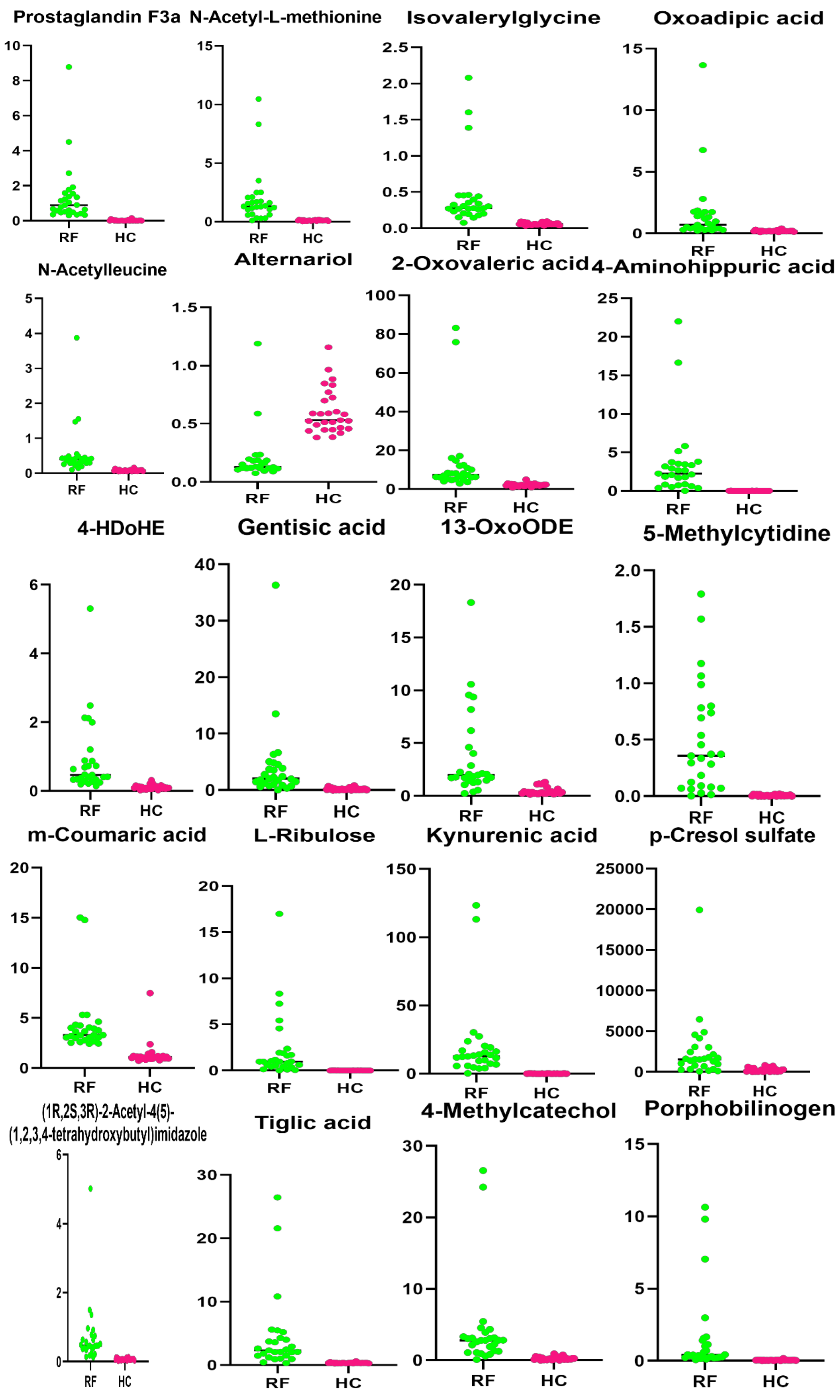
Prostaglandin F3a
N-Acetyl-L-methionine
Isovalerylglycine
Oxoadipic acid
N-Acetylleucine
Alternariol
2-Oxovaleric acid
4-Aminohippuric acid
4-HDoHE
Gentisic acid
13-OxoODE
5-Methylcytidine
m-Coumaric acid
L-Ribulose
Kynurenic acid
p-Cresol sulfate
(1R,2S,3R)-2-Acetyl-4(5)-(1,2,3,4-tetrahydroxybutyl)imidazole
Tiglic acid
4-Methylcatechol
Porphobilinogen
RF
HC

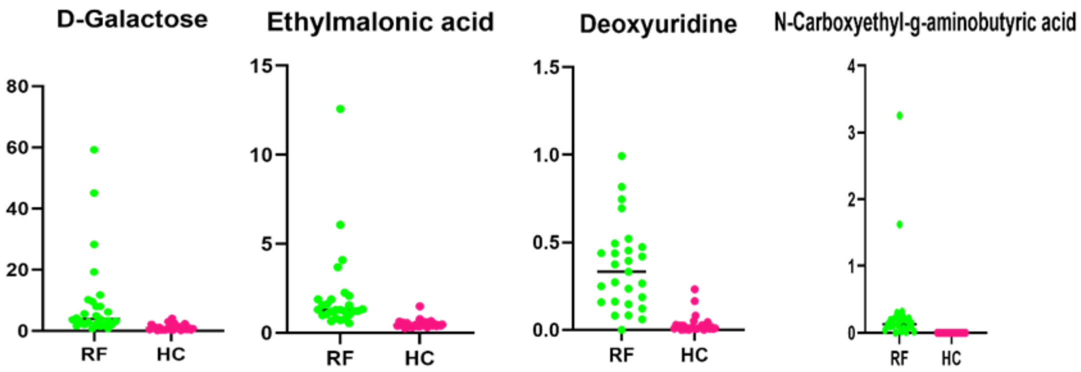

Supplementary Figure S3 Dot map of all the different endogenous metabolites
