# Supplementary material for: Serum Biomarkers for Chronic Renal Failure Screening and Mechanistic Understanding: A Global LC-MS-Based Metabolomics Research
Source: Evid Based Complement Alternat Med. 2022 Jul 30;2022:7450977. doi: 10.1155/2022/7450977 (PMC9356786; doi:10.1155/2022/7450977)
Supplement: Supplementary Materials — Supplementary Figure S1: Quality control diagram. A and B are TIC diagrams of QC samples; C and D are EIC diagrams of internal standard in QC sample; E and F are PCA analysis of QC samples. Supplementary Figure S2: Correlation analysis heat map in positive and negative modes, respectively. Supplementary Figure S3: Dot map of all the different endogenous metabolites. Supplementary Figure S4: Heatmap of hierarchical clustering analysis of group RF vs HC. Supplementary Figure S5: Metabolic pathways with red/blue dots representing the differentially expressed compounds. Red means up regulation, blue means down regulation. Supplementary Figure S6: a KEGG metabolic pathway, Arginine and Proline metabolism. Supplementary Figure S6: b KEGG metabolic pathway, Sphingolipid metabolism. Supplementary Figure S6: c KEGG metabolic pathway, Glycerophospholipid metabolism. Supplementary Figure S6: d KEGG metabolic pathway, D-Arginine and D-ornithine metabolism. Supplementary Figure S7: a KEGG metabolic pathway, Phenylalanine metabolism. Supplementary Figure S7: b KEGG metabolic pathway, Ascorbate and aldarate metabolism. Supplementary Figure S7: c KEGG metabolic pathway, D-Glutamine and D-glutamate metabolism. Supplementary Figure S7: d KEGG metabolic pathway, Arginine and proline metabolism. Supplementary Figure S8: The typical mass spectra of metabolites. Supplementary Table S1: The clinical characteristics of patients. Supplementary Table S2: POS-Differentially Expressed Metabolites. Supplementary Table S3: NEG-Differentially Expressed Metabolites. [file 7450977.f1.zip › Supplementary Table S2.pdf]

Supplementary Table S2 POS-Differentially Expressed Metabolites

Id	Name	MS/MS score	rt	mz	Compounds category	Type	Mean±SD RF	Mean±SD HC	P value
1	Creatinine	0.999969538	197.647	114.0663533	Organic acids and derivatives	forward	62.6453±38.1892	3.3023±1.7316	1.47035E-08
3	Hypoxanthine	0.999943077	231.806	137.0457252	Organoheterocyclic compounds	forward	8.1169±7.7189	0.0055±0.0089	1.00141E-05
6	Choline	0.999865	298.418	104.107117	Organic nitrogen compounds	forward	165.9829±93.1523	35.4341±6.2460	9.70026E-08
8	2-Hydroxypyridine	0.999793231	25.995	96.04468622	Organoheterocyclic compounds	forward	0.9418±1.0144	0.0216±0.0120	7.16853E-05
12	1-Methylhypoxanthine	0.999244462	151.843	151.0614139	Organoheterocyclic compounds	forward	9.7594±7.1060	0.1111±0.0456	1.7159E-07
14	5-Amino-3-oxohexanoate	0.999145615	321.109	146.0810179	Organic acids and derivatives	forward	19.7962±15.3043	1.2442±0.9614	1.14017E-06
16	Dihydrothymine	0.998809846	567.1965	129.0658873	Organoheterocyclic compounds	forward	0.3415±0.1926	1.0360±0.3031	5.65306E-13
18	Creatine	0.998654154	368.7975	132.0765023	Organic acids and derivatives	forward	43.4846±32.0161	10.8373±4.7920	1.57013E-05
19	(1S,2R,4R)-p-Menth-8-ene-2,10-diol 2-glucoside	0.998558692	52.0035	333.1877925	Lipids and lipid-like molecules	forward	0.1010±0.0700	0.0259±0.0125	7.63422E-06
20	Kanzonol Z	0.998542923	280.088	407.1885392	Phenylpropanoids and polyketides	forward	0.3719±0.8633	0.0002±0.0005	0.034064105
21	Beta-Carboline	0.998464923	46.36055	169.0758514	Organoheterocyclic compounds	forward	2.0923±1.8047	0.0959±0.0412	4.72656E-06
22	1H-Indole-3-carboxaldehyde	0.998150231	64.2175	146.0599681	Organoheterocyclic compounds	forward	0.8532±0.3871	0.2599±0.1102	1.46612E-08
23	L-Gulose	0.998041923	317.1425	203.0526593	Organic oxygen compounds	forward	17.0451±12.1921	5.0795±1.5177	2.63849E-05
27	Calystegin A3	0.997597	313.111	160.0967309	Alkaloids and derivatives	forward	32.4756±34.7452	0.4650±1.0691	5.90317E-05
29	5-Acetamidovalerate	0.996949615	353.045	160.0969509	Lipids and lipid-like molecules	forward	1.1103±0.7789	0.04351±0.0376	1.46637E-07
30	Pyroglutamic acid	0.996741615	239.754	130.0498999	Organic acids and derivatives	forward	6.1178±14.8711	0.0172±0.0202	0.042655975
31	Cytosine	0.99649	196.609	112.0587279	Organoheterocyclic compounds	forward	0.7454±0.6454	0.1319±0.0678	4.01689E-05
32	L-Carnitine	0.996317	374.202	162.1122538	Organic nitrogen compounds	forward	508.5629±339.5845	103.2507±28.1549	1.45923E-06
33	4-Hydroxyproline	0.996083538	363.4865	132.065495	Organic acids and derivatives	forward	2.0797±3.0479	0.0864±0.0644	0.002196966
36	Arabinofuranobiose	0.995238462	151.8195	283.1035084	Organic oxygen compounds	forward	2.7744±2.0086	0.0256±0.0150	1.49604E-07
38	p-Aminobenzoic acid	0.994723769	305.627	138.0548812	Benzenoids	forward	27.4879±19.3348	1.0111±0.8764	1.47077E-07
41	Sphinganine	0.992535769	58.9722	302.3049255	Organic nitrogen compounds	forward	0.5643±0.2725	0.1745±0.0474	6.24301E-08
42	Nervonyl carnitine	0.991780615	194.011	102.128017	Organic nitrogen compounds	forward	7.5700±3.8945	2.4425±1.080	2.70815E-07

43	4-Aminophenol	0.991548385	25.8949	110.0602396	Benzenoids	forward	1.67654±1.7464	0.0206±0.0115	4.07933E-05
44	Valdecoxib	0.991139538	448.539	315.0789185	Benzenoids	forward	0.0050±0.0044	7.7438±3.2008	1.50639E-12
45	Kynurenic acid	0.990948538	198.649	190.0496311	Organoheterocyclic compounds	forward	3.1940±2.2843	0.0199±0.0096	1.14558E-07
46	Proline betaine	0.990929	290.579	144.1017526	Organic acids and derivatives	forward	90.7481±91.2462	3.0975±4.61088	3.44981E-05
47	Squamolone	0.990131923	380.399	129.0658323	Organoheterocyclic compounds	forward	12.4707±6.5257	26.1374±9.0989	5.52601E-08
48	Glycerol tripropanoate	0.990047538	61.6063	261.1308308	Lipids and lipid-like molecules	forward	1.3341±0.9809	0.06405±0.0211	3.86613E-07
52	1-Methyladenosine	0.988582615	309.867	282.1192069	Nucleosides, nucleotides, and	forward	2.6741±1.7049	0.4820±0.0746	4.33831E-07
55	Pyrrolidine	0.985695923	560.5405	72.08122247	analogues	forward	0.0981±0.0528	0.0282±0.0137	2.46993E-07
56	L-Methionine	0.985005615	100.867	150.0582155	Organoheterocyclic compounds	forward	4.6009±3.5915	0.1731±0.0754	8.70487E-07
58	Trimethylamine N-oxide	0.983801154	348.8705	76.07611785	Organic acids and derivatives	forward	49.3798±48.7264	1.6738±0.6915	2.66982E-05
59	2-Methylbutyroylcarnitine	0.983716769	264.913	246.1695741	Organic nitrogen compounds	forward	15.9256±16.9229	0.8235±0.4122	8.77139E-05
60	2-Aminoisobutyric acid	0.982295692	318.5965	104.070875	Lipids and lipid-like molecules	forward	5.9953±3.5696	1.2299±0.3968	2.23845E-07
62	4-Guanidinobutanoic acid	0.981638769	343.171	146.0922881	Organic acids and derivatives	forward	11.4722±11.0449	0.0236±0.0093	1.21679E-05
63	Butyrylcarnitine	0.980054769	281.751	232.1539006	Organic acids and derivatives	forward	54.8944±52.8914	1.3352±0.4707	1.68654E-05
66	L-Acetylcarnitine	0.976186846	356.116	204.1229291	Lipids and lipid-like molecules	forward	15.4986±19.7901	0.8386±0.3949	0.000692619
67	1,3-Dihydro-(2H)-indol-2-one	0.976168923	26.8695	134.0599075	Lipids and lipid-like molecules	forward	33.1507±22.8839	1.54680±0.9171	1.2688E-07
68	Oleamide	0.975016923	89.8033	282.278748	Organoheterocyclic compounds	forward	0.3047±0.2789	1.6708±0.7366	1.8858E-10
69	Arecaidine	0.973018692	259.133	142.086218	Lipids and lipid-like molecules	forward	2.2341±1.5630	0.4109±0.4511	2.20606E-06
70	Benzenepropanenitrile	0.972967692	34.46345	132.080716	Alkaloids and derivatives	forward	1.6029±1.5431	0.0864±0.0281	2.53858E-05
75	Cholesterol	0.969985077	31.19195	369.3511847	Benzenoids	forward	9.5621±4.4075	3.1963±2.1949	5.82892E-08
79	Trimethylaminoacetone	0.967917	231.915	116.1071086	Lipids and lipid-like molecules	forward	11.8087±11.5135	0.178±0.0792	1.74382E-05
80	L-Arginine	0.967203	540.655	175.1188883	Organic oxygen compounds	forward	33.4854±24.3564	5.6265±2.4171	2.84708E-06
83	Phytosphingosine	0.965923769	62.4765	318.2994901	Organic acids and derivatives	forward	3.8187±1.8790	1.8811±0.5289	1.48006E-05
86	Valerylglycine	0.964142692	331.5195	160.0967553	Organic nitrogen compounds	forward	15.0035±33.6056	0.1733±0.4697	0.030192019
88	Phenylacetylglutamine	0.962959615	239.041	265.1180444	Organic acids and derivatives	forward	40.1505±83.6683	0.1649±0.1318	0.019791919
89	2-Methylbutyrylglycine	0.960831692	388.414	160.1077736	Organic acids and derivatives	forward	1.8668±1.6067	0.0698±0.0512	4.00887E-06

91	Alcophosphamide	0.959123231	419.298	279.0434222	Organic acids and derivatives	forward	0.0044±0.0037	0.1939±0.0569	7.80443E-16
94	Mesalazine	0.958199692	81.6893	154.0497128	Organic nitrogen compounds	forward	1.7725±1.1173	0.0826±0.0400	2.45235E-08
98	3-Amino-2-piperidone	0.955154692	214.34	115.08674	Benzenoids	forward	1.6649±1.2514	0.1369±0.0591	1.01677E-06
106	Sphingosine	0.944602846	89.807	300.2890586	Organic acids and derivatives	forward	0.2221±0.2123	1.3019±0.5788	1.68227E-10
107	Glycyl-Valine	0.944583923	320.919	175.1078762	Organic nitrogen compounds	forward	0.4861±0.4893	0.0108±0.0070	2.96888E-05
113	apo-[3-methylcrotonoyl-CoA:carbon-dioxide ligase (ADP-forming)]	0.938950615	333.391	174.1235936	Organic acids and derivatives	forward	18.8000±13.6084	0.1263±0.0654	1.42659E-07
115	N1-Methyl-4-pyridone-3-carboxamide	0.937488308	80.9673	153.0657936	Organic acids and derivatives	forward	20.2149±12.5615	0.8799±0.5477	1.76003E-08
116	1,2,3,4,Tetrahydro-1,5,7-trimethylnapthalene	0.937122308	32.3227	175.1480908	Organoheterocyclic compounds	forward	0.4951±0.2807	0.1486±0.0647	8.40263E-07
117	PE(22:5(7Z,10Z,13Z,16Z,19Z)/22:5(7Z,10Z,13Z,16Z,19Z))	0.935284308	73.0161	840.550462	Benzenoids	forward	0.0137±0.0178	0.00008±0.00007	0.000515099
121	4-Butyloxazole	0.930160615	65.99975	126.0913317	Lipids and lipid-like molecules	forward	7.1825±5.4397	0.5517±0.2428	1.04526E-06
122	Glycerophosphocholine	0.929522846	407.557	258.1099863	Organoheterocyclic compounds	forward	0.7847±0.5198	2.6337±1.6552	4.56268E-06
123	Indole-3-carboxylic acid	0.928407923	341.3865	254.1387041	Lipids and lipid-like molecules	forward	1.6743±2.4491	0.0032±0.0112	0.001511174
124	Acetaminophen	0.925664	23.6662	152.0704764	Organoheterocyclic compounds	forward	1.2494±2.4289	0.0153±0.0210	0.013833322
126	Phenylalanyl-Tryptophan	0.925167769	193.5745	352.1653953	Benzenoids	forward	0.0839±0.0538	0.0079±0.0046	8.29766E-08
129	Epidermin	0.924058538	413.784	262.1283947	Organic acids and derivatives	forward	3.5887±2.7069	0.0622±0.0212	3.48986E-07
130	Dodecanoylcarnitine	0.922280077	210.8465	344.2791642	Organic oxygen compounds	forward	4.3788±3.7215	0.4194±0.2462	8.40124E-06
132	Fexofenadine	0.920836154	221.2915	502.291794	Lipids and lipid-like molecules	forward	0.9712±0.6110	0.3223±0.0789	8.72686E-06
133	Leucyl-phenylalanine	0.917413846	191.911	279.1699254	Benzenoids	forward	0.1013±0.0683	0.0143±0.0148	5.0084E-07
135	Dieporeticenin	0.909837308	29.93465	573.488204	Organic acids and derivatives	forward	0.1078±0.0729	0.0388±0.01737	4.76271E-05
136	Dihydrouracil	0.908610077	173.475	115.050369	Lipids and lipid-like molecules	forward	0.7549±0.4824	0.0567±0.0130	5.5207E-08
139	Asymmetric dimethylarginine	0.906649462	520.95	203.1501073	Organoheterocyclic compounds	forward	10.8238±11.8155	1.9855±0.4221	0.000629452
140	4-Pyridoxic acid	0.906070692	42.418	184.0602145	Organic acids and derivatives	forward	2.8718±2.4127	0.0336±0.0318	1.84333E-06
144	1,3-Diisopropylbenzene	0.903669846	32.52695	163.1479117	Organoheterocyclic compounds	forward	0.4457±0.3754	0.0699±0.0223	1.99264E-05
147	5'-Methylthioadenosine	0.899765615	88.98385	298.0965068	Benzenoids	forward	1.1497±0.7839	0.0085±0.0072	4.96926E-08
154	Thelephoric acid	0.893538231	452.5935	353.0261275	Nucleosides, nucleotides, and analogues	forward	0.0275±0.0151	1.6144±0.3074	1.57819E-20
155	Debenzoylzucchini factor B	0.892826077	21.7597	560.4102619	Organoheterocyclic compounds	forward	0.1056±0.0885	0.0020±0.0017	2.01153E-06

157	1-(beta-D-Ribofuranosyl)-1,4-dihydronicotinamide	0.887268077	172.601	257.1130608	Lipids and lipid-like molecules	forward	1.7906±1.2289	0.0576±0.0123	8.82126E-08
158	Prolylhydroxyproline	0.884964385	419.242	229.1182791	Organic oxygen compounds	forward	1.2930±2.7288	0.0243±0.0196	0.023031622
161	3-Methylglutarylcarnitine	0.881474308	409.1975	290.1592506	Organic acids and derivatives	forward	9.3477±8.8526	0.0383±0.0223	9.91234E-06
166	Propionylcarnitine	0.874978	302.855	218.1386668	Lipids and lipid-like molecules	forward	31.2218±38.7763	1.9898±0.7906	0.000580565
168	3-Hydroxyadipic acid 3,6-lactone	0.868650154	327.199	145.0494813	Lipids and lipid-like molecules	forward	1.0159±0.8516	0.1178±0.0372	9.52878E-06
169	1,2-Dihydro-1,1,6-trimethylnaphthalene	0.868534462	32.2344	173.132508	Organoheterocyclic compounds	forward	0.4270±0.3286	0.0868±0.0334	1.24781E-05
171	Triethanolamine	0.866057308	310.637	150.1123655	Benzenoids	forward	0.4057±0.2569	0.1279±0.0349	6.68829E-06
173	PI(20:2(11Z,14Z)/16:0)	0.861782	214.24	863.5635419	Organonitrogen compounds	forward	0.1090±0.0603	0.0422±0.0136	4.74244E-06
175	PC(20:5(5Z,8Z,11Z,14Z,17Z)/20:5(5Z,8Z,11Z,14Z,17Z))	0.852291154	130.037	826.5433722	Lipids and lipid-like molecules	forward	0.3035±0.2091	0.0055±0.0069	7.29236E-08
178	PC(20:5(5Z,8Z,11Z,14Z,17Z)/15:0)	0.850635769	171.703	766.5399111	Lipids and lipid-like molecules	forward	0.1793±0.1181	0.0677±0.0271	4.71775E-05
179	L-alpha-Aspartyl-L-hydroxyproline	0.848212846	332.431	247.0922513	Lipids and lipid-like molecules	forward	5.2223±7.8744	0.0082±0.0046	0.001971604
182	2,3,4,5,6,7-Hexahydro-7-methylcyclopent[b]azepin-8(1H)-one	0.845589615	32.9924	166.1225387	Organic acids and derivatives	forward	0.1448±0.0598	0.0555±0.0336	3.68635E-08
183	2-Methoxy-3-methylpyrazine	0.843267077	196.181	125.0710273	Organoheterocyclic compounds	forward	4.9834±3.2403	0.1592±0.1377	3.27375E-08
185	Imidazole-4-acetaldehyde	0.839591923	154.48	111.0555005	Organoheterocyclic compounds	forward	16.8059±9.2279	3.7029±1.4625	7.22268E-08
186	Valyl-Valine	0.836569	408.76	217.1545372	Organoheterocyclic compounds	forward	5.3979±6.1712	0.0347±0.0341	0.00012069
187	Hexadecanedioic acid mono-L-carnitine ester	0.835499846	293.2875	430.3162211	Organic acids and derivatives	forward	0.0382±0.0235	0.0084±0.0045	5.79406E-07
191	Polyvidone	0.830580846	321.9	178.0533689	Lipids and lipid-like molecules	forward	0.8931±1.1811	0.0204±0.0199	0.000710725
192	Solacauline	0.830380385	149.3595	824.4852932	Organoheterocyclic compounds	forward	1.1408±0.8756	0.0010±0.0026	3.53556E-07
195	Formiminoglutamic acid	0.822003923	230.206	175.0712006	Lipids and lipid-like molecules	forward	16.8531±17.6877	0.1341±0.0618	4.24387E-05
196	2-Methoxy-5-methylpyrazine	0.820804	154.43	125.0710248	Organic acids and derivatives	forward	11.1209±8.3578	0.5584±0.4215	5.77654E-07
197	3-Hydroxyisovalerylcarnitine	0.820742077	327.194	262.1649273	Organoheterocyclic compounds	forward	1.4739±1.2481	0.1032±0.0440	5.27452E-06
198	DG(20:5(5Z,8Z,11Z,14Z,17Z)/16:1(9Z)/0:0)	0.820288923	30.2999	613.4821299	Lipids and lipid-like molecules	forward	0.2532±0.2051	0.0271±0.0240	5.00525E-06
200	Mandelonitrile	0.817842385	46.80745	134.0633193	Lipids and lipid-like molecules	forward	0.3212±0.3813	0.0124±0.0028	0.00027189
203	Acetone cyanohydrin	0.807302615	74.81825	86.0604338	Benzenoids	forward	19.0787±20.5389	3.4354±2.6632	0.000543368
206	PI(18:1(9Z)/18:1(9Z))	0.799433231	214.079	880.5912754	Organic oxygen compounds	forward	0.2485±0.1430	0.0958±0.0277	8.24678E-06
210	Presqualene diphosphate	0.796352769	96.58545	587.3276108	Lipids and lipid-like molecules	forward	0.1123±0.0668	0.00007±0.00002	3.27575E-09

212	Methylimidazole acetaldehyde	0.79346	68.53755	125.0709393	Lipids and lipid-like molecules	forward	35.6273±28.4829	3.0957±3.2743	2.9124E-06
217	beta-Solamarine	0.782912923	159.623	868.5093893	Organoheterocyclic compounds	forward	0.7165±0.5372	0.0022±0.0040	2.46162E-07
219	Lycoperoside D	0.778368615	150.078	740.4640076	Lipids and lipid-like molecules	forward	2.6521±2.0734	0.0108±0.01679	5.08007E-07
220	Pi-Methylimidazoleacetic acid	0.772390231	319.2575	141.0657832	Lipids and lipid-like molecules	forward	36.7091±29.3553	2.3012±1.3408	1.94842E-06
221	1,5-Octadien-3-one	0.771686231	33.07405	125.0961161	Organoheterocyclic compounds	forward	0.1530±0.1069	0.0361±0.0130	5.70229E-06
228	2,6-Dimethoxyphenol	0.762194846	24.2198	155.0701605	Organooxygen compounds	forward	0.4727±0.5641	0.0066±0.0100	0.00021731
232	Indole	0.758970615	33.9726	118.0652031	Benzenoids	forward	1.7381±1.1780	0.5817±0.1162	2.60946E-05
233	Stigmastane-3,6-dione	0.757653308	31.0681	429.3725012	Organoheterocyclic compounds	forward	16.5686±14.8412	4.9851±1.3773	0.000412491
237	N-Salicyloylaspartic acid	0.741836077	180.491	254.0650293	Lipids and lipid-like molecules	forward	0.7498±0.3429	0.2398±0.0549	3.04679E-08
241	PE(16:0/18:2(9Z,12Z))	0.731685	172.601	716.5238035	Organic acids and derivatives	forward	1.7362±1.6711	0.4802±0.2676	0.000636606
256	Betulin	0.698096	30.3521	445.3672843	Lipids and lipid-like molecules	forward	5.2798±6.4269	1.3868±0.4361	0.004148721
258	Tromethamine	0.695797385	38.0412	122.081242	Lipids and lipid-like molecules	forward	0.6980±0.4110	0.2217±0.1693	2.99643E-06
263	2-Oxoarginine	0.685646538	456.0765	174.0872789	Organic compounds	forward	0.3542±0.2769	0.0689±0.0281	1.3416E-05
264	SM(d18:1/16:0)	0.685430846	206.421	703.572257	Organic acids and derivatives	forward	42.4989±23.0571	18.3941±3.6942	1.10499E-05
274	Guanidinosuccinic acid	0.665936846	415.688	176.0666241	Lipids and lipid-like molecules	forward	2.5228±2.0134	0.0359±0.01258	8.44878E-07
276	2-O-(6-Phospho-alpha-mannosyl)-D-glycerate	0.664524769	450.985	349.0548178	Organic acids and derivatives	forward	0.0010±0.0041	0.4733±0.0878	5.61606E-21
280	PC(20:1(11Z)/14:1(9Z))	0.659087846	169.148	758.5686337	Organic oxygen compounds	forward	183.9086±111.1022	78.3913±20.8059	4.23617E-05
282	PC(P-18:0/16:0)	0.656464692	167.428	746.6040151	Lipids and lipid-like molecules	forward	1.3078±0.8573	0.4087±0.1784	1.08192E-05
293	2,8-Di-O-methylellagic acid	0.645040231	453.0945	331.0445455	Lipids and lipid-like molecules	forward	0.2128±0.1077	45.8545±7.7392	6.11502E-22
311	Palmitoleoyl Ethanolamide	0.590239308	93.0331	298.273569	Phenylpropanoids and polyketides	forward	0.0425±0.0543	0.2007±0.0874	4.60803E-10
313	(E)-2,6-Dimethyl-2,5-heptadienoic acid	0.588392154	38.2678	155.1064789	Organic nitrogen compounds	forward	1.0238±0.5500	0.2104±0.0968	3.33011E-08
320	Perlolyrine	0.578192538	26.1126	265.0970253	Lipids and lipid-like molecules	forward	0.5729±0.5112	0.0078±0.0042	4.77709E-06
322	Diethanolamine	0.568962846	325.453	106.0865129	Alkaloids and derivatives	forward	8.0195±4.6504	0.1692±0.0639	3.01183E-09
325	Benzyl methyl sulfide	0.564159462	56.3957	128.106979	Organic nitrogen compounds	forward	0.2291±0.2979	0.0273±0.0110	0.001615105
332	(R)-Pelletierine	0.540067769	33.7287	142.122569	Benzenoids	forward	0.2454±0.1450	0.0793±0.0215	2.81646E-06
333	PC(15:0/18:2(9Z,12Z))	0.534705846	171.7015	744.5542303	Organoheterocyclic compounds	forward	2.7649±1.8128	0.9941±0.4200	3.01301E-05

336	3,3',4',5,6,7,8-Heptahydroxyflavone	0.526300846	451.266	335.0372042	Phenylpropanoids and polyketides	forward	0.0026±0.0018	1.0119±0.1752	1.11708E-21
337	2,6 Dimethylheptanoyl carnitine	0.523058385	222.151	302.2319952	Lipids and lipid-like molecules	forward	2.6087±2.5289	0.1365±0.1401	2.73428E-05
338	Propionylglycine	0.522073308	450.77	132.0654846	Organic acids and derivatives	forward	0.1381±0.3035	1.1950±0.2059	1.69576E-20
341	SM(d18:1/24:1(15Z))	0.505571077	200.475	813.6823494	Lipids and lipid-like molecules	forward	11.3516±6.8049	4.5310±1.3373	2.04988E-05
343	Deoxycholic acid	0.501135538	49.3562	415.27956	Lipids and lipid-like molecules	forward	0.1859±0.1326	0.0275±0.0164	1.44375E-06
345	Hexylamine	0.5	37.2255	102.127991	Organonitrogen compounds	reverse	2.1881±1.1162	0.9169±0.1843	3.06627E-06
349	Threoninyl-Aspartate	0.493626154	419.6955	235.0922903	Organic acids and derivatives	forward	0.0348±0.0166	2.1466±0.6628	2.4657E-15
352	L-beta-aspartyl-L-threonine	0.486760615	435.072	235.0920537	Organic acids and derivatives	forward	0.3303±0.1245	1.0874±0.2464	6.28873E-17
353	4,8 Dimethylnonanoyl carnitine	0.479288077	210.858	330.2634116	Lipids and lipid-like molecules	forward	1.6712±1.8055	0.2994±0.4341	0.000619022
359	Serylalanine	0.458556462	435.2755	177.0869604	Organic acids and derivatives	forward	0.0719±0.027	0.4607±0.2203	1.09853E-09
360	Isoleucyl-Alanine	0.455819923	374.201	203.1390883	Organic acids and derivatives	forward	4.8835±4.5489	0.2360±0.0819	1.49119E-05
361	5,6-Dihydrouridine	0.455314769	173.4685	247.092148	Organic oxygen compounds	forward	1.6309±1.0959	0.0645±0.0145	6.92302E-08
363	Paraquat dichloride	0.437763923	419.2985	257.0615716	Organic compounds	forward	0.0542±0.0326	0.8432±0.2406	7.35496E-16
366	Santene	0.402324077	33.0336	123.1168429	Hydrocarbons	forward	0.2146±0.1175	0.0621±0.0164	3.64457E-07

MS/MS score: the matching score of mass spectrometry secondary ion, ranged from 0 to 1, and the greater the value, the better; Compounds category: classification information of the substance in HMDB database. Mean±SD RF: Mean, the mean of the relative quantitative value of the RF group; SD, the standard deviation of the relative quantitative value of the RF group; Mean±SD HC: Mean, the mean of the relative quantitative value of the HC group; SD, the standard deviation of the relative quantitative value of the HC group.
