# Supplementary material for: Serum Biomarkers for Chronic Renal Failure Screening and Mechanistic Understanding: A Global LC-MS-Based Metabolomics Research
Source: Evid Based Complement Alternat Med. 2022 Jul 30;2022:7450977. doi: 10.1155/2022/7450977 (PMC9356786; doi:10.1155/2022/7450977)
Supplement: Supplementary Materials — Supplementary Figure S1: Quality control diagram. A and B are TIC diagrams of QC samples; C and D are EIC diagrams of internal standard in QC sample; E and F are PCA analysis of QC samples. Supplementary Figure S2: Correlation analysis heat map in positive and negative modes, respectively. Supplementary Figure S3: Dot map of all the different endogenous metabolites. Supplementary Figure S4: Heatmap of hierarchical clustering analysis of group RF vs HC. Supplementary Figure S5: Metabolic pathways with red/blue dots representing the differentially expressed compounds. Red means up regulation, blue means down regulation. Supplementary Figure S6: a KEGG metabolic pathway, Arginine and Proline metabolism. Supplementary Figure S6: b KEGG metabolic pathway, Sphingolipid metabolism. Supplementary Figure S6: c KEGG metabolic pathway, Glycerophospholipid metabolism. Supplementary Figure S6: d KEGG metabolic pathway, D-Arginine and D-ornithine metabolism. Supplementary Figure S7: a KEGG metabolic pathway, Phenylalanine metabolism. Supplementary Figure S7: b KEGG metabolic pathway, Ascorbate and aldarate metabolism. Supplementary Figure S7: c KEGG metabolic pathway, D-Glutamine and D-glutamate metabolism. Supplementary Figure S7: d KEGG metabolic pathway, Arginine and proline metabolism. Supplementary Figure S8: The typical mass spectra of metabolites. Supplementary Table S1: The clinical characteristics of patients. Supplementary Table S2: POS-Differentially Expressed Metabolites. Supplementary Table S3: NEG-Differentially Expressed Metabolites. [file 7450977.f1.zip › Supplementary Table S3.pdf]

Supplementary Table S3 NEG-Differentially Expressed Metabolites

Id	Name	MS/MS score	rt	mz	Compounds category	Type	Mean±SD RF	Mean±SD HC	P value
3	Caprylic acid	1	54.0216	143.1072723	Lipids and lipid-like molecules	forward	14.0075±10.5521	6.33020±1.2279	0.000854085
7	Propionic acid	1	70.3274	73.02881477	Organic acids and derivatives	forward	1.4592±1.4274	0.5849±0.2235	0.00399301
15	Pyrrolidonecarboxylic acid	0.999994231	311.661	128.0347043	Organic acids and derivatives	forward	153.7552±203.9665	31.7260±6.6955	0.004524188
22	Saccharin	0.999884923	30.33525	181.9914839	Organoheterocyclic compounds	forward	35.5476±73.1546	0.5352±1.0511	0.019637872
24	Creatinine	0.999678538	179.684	112.0511144	Organic acids and derivatives	forward	22.4240±30.3281	0.9818±0.3125	0.001088507
27	4-Hydroxybenzaldehyde	0.999200308	38.0442	121.0289681	Organic oxygen compounds	forward	9.2620±7.5247	0.7132±0.5618	3.15974E-06
28	Cyclamic acid	0.999189077	59.30015	178.0509683	Organic acids and derivatives	forward	91.5275±160.8318	1.1328±2.7102	0.007137329
29	N-Acetyl-L-alanine	0.999152615	265.32	130.0504718	Organic acids and derivatives	forward	6.8871±9.3570	0.3387±0.0689	0.001197553
31	4-Hydroxyproline	0.998849923	364.003	130.0504878	Organic acids and derivatives	forward	8.4562±11.4743	0.5688±0.3534	0.001415979
33	Capric acid	0.998748615	218.306	171.1387503	Lipids and lipid-like molecules	forward	1.0963±1.3247	0.1214±0.0472	0.000740782
34	Orotidine	0.998599462	319.565	287.0526097	Nucleosides, nucleotides, and analogues	forward	7.8546±17.9029	0.0966±0.0307	0.03301565
35	4-Hydroxybenzoic acid	0.998473769	223.68	137.0239754	Benzenoids	forward	8.1400±16.2311	0.0519±0.0179	0.015548261
37	Glutaric acid	0.998056385	205.239	131.0345201	Organic acids and derivatives	forward	1.9834±2.7253	0.4566±0.2156	0.007402282
39	Adenine	0.997691769	363.0865	134.0467175	Organoheterocyclic compounds	forward	0.1654±0.2221	0.5866±0.2954	2.5684E-07
40	Pyrocatechol	0.997579385	23.0624	109.0288867	Benzenoids	forward	10.2551±18.3376	0.9812±0.5915	0.0142581
53	Indoxyl sulfate	0.993766	26.7344	212.0021843	Organic acids and derivatives	forward	4362.1784±5319.7127	121.2649±89.8552	0.000322399
58	N-Acetylarylamine	0.992374462	204.614	134.060705	Benzenoids	forward	29.5256±54.9024	0.4661±0.4781	0.010694928
66	Ribothymidine	0.988856692	144.583	257.0782665	Nucleosides, nucleotides, and analogues	forward	1.6410±1.6240	0.5137±0.0918	0.001302149
71	4-Hydroxyhippuric acid	0.985692077	252.7815	194.0459171	Benzenoids	forward	10.1686±20.0940	0.0177±0.0140	0.014321569
74	Isobutyrylglycine	0.983696385	221.023	144.0661503	Organic acids and derivatives	forward	1.5327±1.9181	0.2532±0.4603	0.002139951
78	2-Hydroxyethanesulfonate	0.982715615	151.5265	124.9908861	Organic acids and derivatives	forward	434.9390±619.2727	6.5652±3.7340	0.001333737
80	D-Proline	0.982009538	328.612	114.0554811	Organic acids and derivatives	forward	40.3084±63.6525	7.7153±2.8221	0.013241179
82	Pelargonic acid	0.980508231	50.7365	157.1230139	Lipids and lipid-like molecules	forward	71.0479±60.680	32.3948±5.0998	0.002787282
88	p-Hydroxyphenylacetic acid	0.977393846	36.80355	151.0396777	Benzenoids	forward	4.1280±5.2440	0.1419±0.1075	0.000533522

89	Mycophenolic acid	0.975560769	319.8425	319.1151455	Organoheterocyclic compounds	forward	1.0404±1.0286	0.0071±0.0077	1.88344E-05
91	Uracil	0.974649923	319.5715	111.0194725	Organoheterocyclic compounds	forward	5.2806±11.1595	0.1373±0.0318	0.024124172
98	Salicyluric acid	0.967236615	180.1635	194.0458117	Benzenoids	forward	97.1913±212.4084	0.9352±0.3089	0.026365926
99	Formylanthranilic acid	0.966295077	77.9509	164.034993	Benzenoids	forward	24.6067±27.3537	0.2886±0.2076	9.1784E-05
101	Phenylacetylglutamine	0.965609077	239.402	263.1038646	Organic acids and derivatives	forward	112.0520±166.9128	0.3831±0.3036	0.001801148
104	Phenyllactic acid	0.962355769	113.485	165.0553624	Phenylpropanoids and polyketides	forward	13.3130±11.6874	2.3325±1.0186	4.64537E-05
107	Trehalose	0.959447385	150.8685	341.1111021	Organic oxygen compounds	forward	2.0810±2.1092	0.0239±0.0109	2.8119E-05
109	Hydantoin-5-propionic acid	0.954046769	310.633	171.0408434	Organoheterocyclic compounds	forward	5.3010±10.3886	0.0065±0.0113	0.013572675
113	3-Hydroxymethylglutaric acid	0.951970923	380.7545	161.0452287	Lipids and lipid-like molecules	forward	14.4711±19.5950	0.6515±0.1865	0.001114084
115	(10E,12Z)-9-HODE	0.950906	52.2063	295.2283823	Lipids and lipid-like molecules	forward	12.2174±10.7725	1.4717±0.5413	2.07907E-05
116	D-Ribose	0.950161154	112.68	149.0451724	Organic oxygen compounds	forward	9.4928±10.0401	2.8547±0.8268	0.002030929
117	Threonic acid	0.949760615	387.074	135.0294249	Organic oxygen compounds	forward	2.9705±5.0206	0.1848±0.0760	0.007804981
120	(2E)-Decenoyl-ACP	0.948655846	289.6765	128.0712321	Organic acids and derivatives	forward	0.1384±0.4043	0.5791±0.4856	0.000660183
123	Inosine	0.945832077	231.619	267.0737644	Nucleosides, nucleotides, and analogues	forward	17.4400±28.2917	0.0183±0.0164	0.003605248
126	L-Glutamic acid	0.940484692	94.2287	146.0454633	Organic acids and derivatives	forward	1.6275±1.4434	0.3991±0.1757	0.000159218
128	Pseudouridine	0.938465308	257.558	243.0625274	Nucleosides, nucleotides, and analogues	forward	26.0124±33.7611	0.8230±0.1890	0.000644041
130	D-Xylitol	0.937086692	249.216	151.0608523	Organic oxygen compounds	forward	11.5080±21.2492	0.2592±0.0943	0.010682405
131	4-Hydroxybenzeneacetonitrile	0.935833769	27.3018	132.0447767	Benzenoids	forward	13.0193±16.1708	0.3735±0.3116	0.000396476
132	D-Glucose	0.935809769	205.083	179.0540062	Organic oxygen compounds	forward	45.3615±81.5061	0.7358±0.7493	0.008547409
134	L-Phenylalanine	0.933939462	277.346	164.0714044	Organic acids and derivatives	forward	33.7420±44.5648	7.9617±1.9043	0.005826468
136	Mesylate	0.929802538	111.8135	94.9801538	Organic compounds	forward	170.5409±301.0233	4.9832±2.3311	0.008289375
137	2-Keto-3-deoxy-D-gluconic acid	0.929642769	309.915	177.0402102	Organic acids and derivatives	forward	2.1074±4.1154	0.1104±0.0618	0.018162048
143	N-Acetyl-L-phenylalanine	0.915208462	193.2355	206.0822434	Organic acids and derivatives	forward	0.68741±0.7403	0.0338±0.0258	0.000100073
145	trans-Aconitic acid	0.913047154	448.127	173.0088935	Organic acids and derivatives	forward	11.5957±12.3767	2.9506±1.7386	0.00127977
147	D-Xylulose	0.907969462	77.8917	149.0452135	Organooxygen compounds	forward	8.9817±5.9651	1.9276±1.8864	1.80331E-06
148	myo-Inositol	0.907463154	408.274	179.0558853	Organic oxygen compounds	forward	35.8111±45.5321	1.1396±0.3376	0.000523363

149	Sucrose	0.906667769	382.605	341.1095278	Organic oxygen compounds	forward	2.3454±5.6310	0.0140±0.0332	0.04091666
150	L-Gulonolactone	0.905635	112.6235	177.0402085	Organoheterocyclic compounds	forward	19.3006±16.7250	6.4178±1.8472	0.000477969
152	4-Pyridoxic acid	0.902442615	41.37695	182.0456347	Organoheterocyclic compounds	forward	12.5414±13.8628	0.1774±0.1275	8.82538E-05
153	L-Gulose	0.902255077	292.276	179.056023	Organic oxygen compounds	forward	2.6010±5.7456	0.1564±0.0592	0.03605426
155	Acetylglycine	0.901349615	293.924	116.0347957	Organic acids and derivatives	forward	4.0823±4.4550	0.3488±0.1250	0.000184867
156	L-Fucose	0.901149769	209.256	163.06086	Organic oxygen compounds	forward	1.4117±1.8615	4.7223±1.3786	1.0369E-09
158	Traumatic acid	0.896350615	319.582	227.1289816	Lipids and lipid-like molecules	forward	1.2521±1.6401	0.0190±0.0211	0.000596425
161	2-Furoylglycine	0.890770077	214.917	168.0300095	Organic acids and derivatives	forward	9.4909±16.1734	0.0185±0.0075	0.005297352
162	L-Tyrosine	0.890371846	317.689	180.0593599	Organic acids and derivatives	forward	3.3980±3.4641	0.5729±0.2192	0.000253155
163	2-Indolecarboxylic acid	0.889196538	73.6544	160.0400847	Organoheterocyclic compounds	forward	0.79471.2276	0.1592±0.0461	0.012359819
166	Azelaic acid	0.888864923	351.762	187.0975316	Lipids and lipid-like molecules	forward	3.2567±4.1809	0.1012±0.0204	0.000573203
167	1,11-Undecanedicarboxylic acid	0.884446615	269.48	243.1603719	Lipids and lipid-like molecules	forward	0.5227±0.9173	0.0490±0.0448	0.012585088
169	N-Acetylglutamine	0.883452769	324.0315	187.0723602	Organic acids and derivatives	forward	0.8132±1.2332	0.0208±0.0092	0.00255037
173	3-Carboxy-4-methyl-5-propyl-2-furanpropionic acid	0.866914308	196.238	239.09275	Lipids and lipid-like molecules	forward	33.0130±56.3256	0.5638±0.5027	0.005980123
178	Citramalic acid	0.861421385	54.62215	147.0294759	Lipids and lipid-like molecules	forward	0.5565±0.3475	0.2336±0.3366	0.001060719
180	Phenol glucuronide	0.853509385	249.1665	269.0551134	Organic oxygen compounds	forward	0.9167±1.5550	0.0104±0.0069	0.005495461
181	5-KETE	0.852146615	50.96285	317.2125195	Lipids and lipid-like molecules	forward	0.8773±0.6706	0.0839±0.0517	1.68073E-06
183	Indolelactic acid	0.849264462	178.803	204.0665959	Organoheterocyclic compounds	forward	16.3144±29.4727	0.8341±0.6268	0.011246574
184	Prolylhydroxyproline	0.849149462	441.313	227.1037545	Organic acids and derivatives	forward	0.6167±1.3131	0.0024±0.0064	0.022252051
187	Hippuric acid	0.833718462	205.021	178.0505746	Benzenoids	forward	457.6307±831.5038	7.7416±7.6823	0.009257289
189	N-Formyl-L-methionine	0.832489615	321.289	176.0384497	Organic acids and derivatives	forward	6.1561±7.5716	0.0574±0.0240	0.000287874
190	4-Acetamidobutanoic acid	0.831477769	294.872	144.0660954	Organic acids and derivatives	forward	15.9363±19.5262	0.0207±0.0347	0.000252515
195	Coumesterol	0.824556385	452.3005	267.0297529	Phenylpropanoids and polyketides	forward	0.0005±0.0006	0.1055±0.0534	1.3898E-10
200	1H-Indole-2,3-dione	0.816718077	45.7762	146.0243335	Organoheterocyclic compounds	forward	3.6831±3.8729	1.0852±0.2123	0.001772142
201	2-Furoic acid	0.810629615	358.428	111.0082104	Organoheterocyclic compounds	forward	1.73890±2.6093	0.0418±0.0183	0.002300899
202	Thymine	0.808172923	68.51285	125.0351976	Organoheterocyclic compounds	forward	3.0986±3.5042	0.3648±0.0522	0.00040658

203	D-Glucuronic acid	0.805414846	400.089	193.0353291	Organic oxygen compounds	forward	2.8388±6.0047	0.0435±0.0493	0.022871769
204	3-Hydroxyvalproic acid	0.804987308	93.52445	159.10235	Lipids and lipid-like molecules	forward	6.3962±7.4669	1.2344±0.5658	0.001360389
205	N-Acetylserine	0.803324	309.918	146.0454316	Organic acids and derivatives	forward	38.4367±55.1835	0.8411±0.2355	0.001531984
207	Dihydrolipoate	0.801791462	94.3932	207.0510924	Lipids and lipid-like molecules	forward	3.0306±2.8428	0.5709±0.1821	0.000128204
211	N4-Acetylcytidine	0.780840154	174.062	284.0890143	Nucleosides, nucleotides, and analogues	forward	1.3607±1.7915	0.0872±0.0359	0.001034948
212	Nicotinamide riboside	0.776304615	245.5785	253.0832319	Organic oxygen compounds	forward	0.3263±0.4542	0.0066±0.0038	0.001135285
213	Methyl jasmonate	0.770764385	169.662	223.1340648	Lipids and lipid-like molecules	forward	1.5861±1.4344	0.2125±0.1052	3.61007E-05
215	8-Hydroxyoctanoate	0.766671	153.7645	159.1023366	Organic compounds	forward	4.1808±5.9590	0.2654±0.0918	0.002109319
216	3-Methoxy-4-hydroxyphenylethyleneglycol sulfate	0.760660846	41.2958	263.0235587	Organic acids and derivatives	forward	59.3140±89.437	0.9491±0.3492	0.002235533
218	20-Hydroxyeicosatetraenoic acid	0.758692846	49.9594	319.2283826	Lipids and lipid-like molecules	forward	4.6748±6.1850	0.4076±0.1892	0.001369259
219	N2,N2-Dimethylguanosine	0.757488846	207.081	310.1164226	Nucleosides, nucleotides, and analogues	forward	0.3992±0.4996	0.0009±0.0022	0.00032241
226	Glutamylthreonine	0.729374923	452.1705	247.0935387	Organic acids and derivatives	forward	0.0044±0.0084	3.6741±0.8184	5.99084E-19
229	Aldehydo-D-xylose	0.724959846	201.291	149.0451269	Organic oxygen compounds	forward	2.1313±2.6755	0.2564±0.0740	0.001185028
230	3,7-Dimethyluric acid	0.714108231	358.536	195.0510026	Organoheterocyclic compounds	forward	2.2730±5.2237	0.1561±0.0902	0.045069379
232	N-acetyl-5-aminosalicylic acid	0.702621615	79.25975	194.0494093	Benzenoids	forward	7.7900±15.5046	0.0280±0.0254	0.015122999
234	Prostaglandin J2	0.697589923	94.4153	333.2080196	Lipids and lipid-like molecules	forward	0.6393±0.6577	0.0436±0.0172	7.31875E-05
235	2-Oxovaleric acid	0.694993692	152.433	115.0395049	Organic acids and derivatives	forward	13.4066±19.4429	2.1794±0.7701	0.005900526
236	4-Aminohippuric acid	0.690602615	207.5955	193.0617795	Benzenoids	forward	3.5218±4.8466	0.0129±0.0062	0.000867164
241	Alternariol	0.674308	420.021	257.0449956	Phenylpropanoids and polyketides	forward	0.1966±0.2200	0.6069±0.1934	1.80104E-09
245	N-Acetylleucine	0.658298846	206.781	172.09767	Organic acids and derivatives	forward	0.5636±0.7394	0.0830±0.0280	0.002322385
246	Prostaglandin F3a	0.658201231	95.18765	351.2184315	Lipids and lipid-like molecules	forward	1.3822±1.7308	0.0241±0.0365	0.000382716
247	Isovalerylglycine	0.657408385	221.928	158.0819723	Organic acids and derivatives	forward	0.4417±0.4711	0.0613±0.01788	0.000280904
251	Oxoadipic acid	0.644359923	177.509	159.0407603	Organic acids and derivatives	forward	1.6076±2.7380	0.2071±0.0653	0.013291315
252	N-Acetyl-L-methionine	0.642457077	210.618	190.0541732	Organic acids and derivatives	forward	1.9344±2.3047	0.1060±0.0472	0.000340232
253	4-HDoHE	0.638602923	50.26695	343.2284374	Lipids and lipid-like molecules	forward	0.9157±1.0996	0.1109±0.0624	0.000786005
254	Gentisic acid	0.637071615	62.7092	153.0190259	Benzenoids	forward	4.1548±7.0041	0.1869±0.1854	0.006756662

255	5-Methylcytidine	0.634026538	214.132	256.0942338	Nucleosides, nucleotides, and analogues	forward	0.4944±0.4848	0.0067±0.0061	1.84856E-05
259	13-OxoODE	0.613559385	52.5842	293.212648	Lipids and lipid-like molecules	forward	3.7328±4.1550	0.4800±0.3688	0.000398745
261	m-Coumaric acid	0.606733923	56.55345	163.0396925	Phenylpropanoids and polyketides	forward	4.3589±3.1443	1.3755±1.2617	6.00396E-05
266	L-Ribulose	0.594728923	249.144	149.0451588	Organic oxygen compounds	forward	2.3121±3.6399	0.0139±0.0034	0.002947028
268	L-Malic acid	0.593378385	327.396	133.0138418	Organic acids and derivatives	forward	5.4676±8.8424	0.2679±0.0980	0.005142833
269	p-Cresol sulfate	0.590056462	23.958	187.0066468	Organic acids and derivatives	forward	2520.5273±3832.2555	238.3081±242.2456	0.004718532
270	Kynurenic acid	0.580990769	197.765	188.0352149	Organoheterocyclic compounds	forward	20.5441±29.1195	0.1237±0.0490	0.001174929
273	Porphobilinogen	0.571872615	303.711	225.0879759	Organic nitrogen compounds	forward	1.5883±2.8517	0.0498±0.0262	0.009437797
274	4-Methylcatechol	0.567309846	23.0779	123.0445339	Benzenoids	forward	4.1521±6.2659	0.2504±0.2187	0.003308147
275	Tiglic acid	0.566882385	196.201	99.04455918	Lipids and lipid-like molecules	forward	4.4166±6.0886	0.3558±0.0685	0.001851668
280	(1R,2S,3R)-2-Acetyl-4(5)-(1,2,3,4-tetrahydroxybutyl)imidazole	0.539992308	327.886	229.0832531	Organic oxygen compounds	forward	0.7153±0.9216	0.0711±0.0259	0.001213463
281	N-Carboxyethyl-g-aminobutyric acid	0.513940462	281.528	174.0769472	Organic acids and derivatives	forward	0.3119±0.6597	0.00034599±0.0004	0.021149776
282	Gingerol	0.507238846	94.2896	293.1761804	Benzenoids	forward	1.1140±1.0594	0.4053±0.0996	0.001842839
283	Deoxyuridine	0.505785923	105.575	227.067565	Nucleosides, nucleotides, and analogues	forward	0.3558±0.2467	0.0307±0.0524	2.67818E-07
288	Ethylmalonic acid	0.476818231	317.78	131.0344823	Lipids and lipid-like molecules	forward	2.0699±2.4180	0.4898±0.2318	0.002260281
293	Gallic acid	0.423272308	36.8293	169.0171027	Benzenoids	forward	1.6457±2.6019	0.1084±0.0467	0.004967173
295	D-Galactose	0.304327154	23.0499	229.017633	Organic oxygen compounds	reverse	9.3948±13.8745	1.1651±1.05395	0.00488423

MS/MS score: the matching score of mass spectrometry secondary ion, ranged from 0 to 1, and the greater the value, the better; Compounds category: classification information of the substance in HMDB database. Mean±SD RF: Mean, the mean of the relative quantitative value of the RF group; SD, the standard deviation of the relative quantitative value of the RF group; Mean±SD HC: Mean, the mean of the relative quantitative value of the HC group; SD, the standard deviation of the relative quantitative value of the HC group.
