# Supplementary material for: Serum Biomarkers for Chronic Renal Failure Screening and Mechanistic Understanding: A Global LC-MS-Based Metabolomics Research
Source: Evid Based Complement Alternat Med. 2022 Jul 30;2022:7450977. doi: 10.1155/2022/7450977 (PMC9356786; doi:10.1155/2022/7450977)
Supplement: Supplementary Materials — Supplementary Figure S1: Quality control diagram. A and B are TIC diagrams of QC samples; C and D are EIC diagrams of internal standard in QC sample; E and F are PCA analysis of QC samples. Supplementary Figure S2: Correlation analysis heat map in positive and negative modes, respectively. Supplementary Figure S3: Dot map of all the different endogenous metabolites. Supplementary Figure S4: Heatmap of hierarchical clustering analysis of group RF vs HC. Supplementary Figure S5: Metabolic pathways with red/blue dots representing the differentially expressed compounds. Red means up regulation, blue means down regulation. Supplementary Figure S6: a KEGG metabolic pathway, Arginine and Proline metabolism. Supplementary Figure S6: b KEGG metabolic pathway, Sphingolipid metabolism. Supplementary Figure S6: c KEGG metabolic pathway, Glycerophospholipid metabolism. Supplementary Figure S6: d KEGG metabolic pathway, D-Arginine and D-ornithine metabolism. Supplementary Figure S7: a KEGG metabolic pathway, Phenylalanine metabolism. Supplementary Figure S7: b KEGG metabolic pathway, Ascorbate and aldarate metabolism. Supplementary Figure S7: c KEGG metabolic pathway, D-Glutamine and D-glutamate metabolism. Supplementary Figure S7: d KEGG metabolic pathway, Arginine and proline metabolism. Supplementary Figure S8: The typical mass spectra of metabolites. Supplementary Table S1: The clinical characteristics of patients. Supplementary Table S2: POS-Differentially Expressed Metabolites. Supplementary Table S3: NEG-Differentially Expressed Metabolites. [file 7450977.f1.zip › Supplementary Table S1 (1).pdf]

Number	Gender	Age	Weight	Height	Blood pressure		Original disease
RF1	female	25	46.7	1.58	157	104	Diabetic nephropathy
RF2	male	27	67.5	1.78	130	86	Chronic nephritis
RF3	female	30	79	1.68	141	90	Chronic nephritis
RF4	male	38	72.5	1.75	93	62	Chronic nephritis
RF5	male	35	81.8	1.7	163	107	Chronic nephritis
RF6	male	36	66.8	1.78	121	76	Chronic nephritis
RF7	male	36	65.5	1.78	163	107	Chronic nephritis
RF8	female	33	48.2	1.62	115	87	Chronic nephritis
RF9	male	32	71.8	1.75	156	105	Chronic nephritis
RF10	male	46	79.9	1.7	146	96	Hypertensive Kidney
RF11	male	44	55	1.68	118	73	Chronic nephritis
RF12	male	49	79	1.78	115	81	Chronic nephritis
RF13	male	42	64.3	1.76	148	103	Chronic nephritis
RF14	male	46	70.8	1.7	127	92	Chronic nephritis
RF15	male	45	67.9	1.75	165	111	Chronic nephritis
RF16	male	43	57.9	1.72	190	196	Diabetic nephropathy
RF17	male	48	64.1	1.72	189	100	Diabetic nephropathy
RF18	male	51	76.8	1.76	144	96	Hypertensive Kidney
RF19	male	53	82.1	1.7	114	78	Diabetic nephropathy
RF20	male	51	62.8	1.76	129	68	Chronic nephritis
RF21	male	52	87	1.7	168	101	Diabetic nephropathy
RF22	male	51	70.8	1.76	142	76	Chronic nephritis,Dia[illegible]
RF23	male	54	63.4	1.72	142	81	Polycystic kidney
RF24	male	70	77	1.72	118	70	Chronic nephritis
RF25	male	69	64.7	1.63	129	88	Chronic nephritis
RF26	male	78	43.1	1.75	134	69	Hypertensive Kidney
RF27	female	67	70.2	1.63	113	71	Chronic nephritis
Average		46.33333	68.02222	1.717037	139.63	91.6	
SD		13.36183	11.05961	0.053767	23.838	25.2	

Past medical history	BMI	ALT	AST	AST/ALT	Urea	Crea umol
Coronary heart disease, Rer	18.70694	6.8	18.5	2.7	10.4	690
No past medical history	21.30413	11	10.9	1	23.8	1130.7
No past medical history	27.99036	1.9	7.1	3.7	19.4	1212.5
Renal hypertension	23.67347	9.4	13.5	1.4	26.1	1047.6
Coronary heart disease, Rer	28.3045	10.2	11.9	1.2	17.71	1074
No past medical history	21.0832	6.5	11.6	1.8	30.9	1107
Hypertension	20.67289	21.3	7.3	0.3	18.2	773.6
Coronary heart disease, Rer	18.3661	5.8	13.7	2.4	20.4	832.2
Hypertension	23.4449	36.3	18.8	0.5	18.55	893.1
Coronary heart disease	27.64706	2.5	7.9	3.2	26.2	1532.6
Coronary heart disease	19.48696	13.1	18.8	1.4	14.18	927.1
Coronary heart disease, Rer	24.93372	16	14.1	0.9	16.82	704.2
Renal hypertension	20.75801	7.3	6.1	0.8	23.09	1203.1
Coronary heart disease	24.49827	12.9	12.9	1.1	27.7	1212.2
Renal hypertension	22.17143	35.6	37.7	1.1	13.9	1285.3
Hypertension, Diabetes	19.57139	11.7	6.5	0.6	20.77	807.4
Hypertension, Diabetes	21.66712	8.5	10.8	1.3	25.8	987.1
Renal hypertension	24.79339	14.9	14	0.9	23.7	934.4
Coronary heart disease, Rer	28.4083	12.9	11.4	0.9	22.3	612.7
Coronary heart disease, Hy	20.27376	5.2	13.6	2.6	27.5	1197.8
Coronary heart disease, Hy	30.10381	14.3	16.9	1.2	15.6	927.4
Hypertension, Diabetes	22.8564	9.1	12.2	1.3	16.18	672
Coronary heart disease, Dia	21.4305	7.5	11.1	1.5	14.3	601.8
Renal hypertension	26.02758	12.7	19.8	1.6	14.78	474.1
Coronary heart disease	24.35169	24.4	13.4	0.5	15.56	821.7
Coronary heart disease, Rer	14.07347	6.3	13.3	2.1	19.7	656.5
Renal hypertension	26.42177	3.7	6.2	1.7	25.3	983.5
	23.07486	12.14074	13.33333	1.47037	20.32741	937.0963
	3.720047	8.615472	6.258164	0.839787	5.226973	250.7235

scr mg/dl	eGFR	UA	GLU	TG	TC	HDL-C	LDL-C
7.80543	6.544997	335.2	6.25	0.88	3.14	1.78	1.96
12.79072	4.712939	431.1	4.45	2.07	2.71	0.74	2.71
13.71606	3.19634	447.4	4.76	0.9	2.97	0.91	2.82
11.85068	4.784254	554.2	4.56	1.49	2.43	0.7	1.81
12.14932	4.741313	433.7	3.96	1.38	3.4	1.13	2.69
12.52262	4.538973	527.1	5.56	1.71	2.27	0.97	1.77
8.751131	7.000284	385.6	4.88	1.49	1.84	0.99	1.1
9.414027	4.933064	390.7	4.6	1.53	3.32	0.97	2.6
10.10294	4.561257	883.2	12.61	6.2	4.42	0.58	1.62
17.3371	2.151933	515.5	3.9	2.2	3.21	0.63	2.81
10.48756	5.317041	428.6	6.19	0.7	2.42	1.36	1.49
7.966063	7.158265	458.5	5.43	3.44	4.52	0.74	3.72
13.60973	3.934982	370.6	7.32	0.85	2.8	1.15	2.02
13.71267	3.791257	392.5	3.65	0.72	3.01	1.39	2.66
14.53959	3.557041	537	6.01	1.42	3.56	1.04	3.01
9.133484	6.328583	402.3	13.49	0.6	3.72	1.87	2.63
11.16629	4.79226	426.3	12.48	1.65	3.48	0.78	3.14
10.57014	5.014141	565.9	5.13	3.09	2.6	0.72	2.07
6.930995	8.235436	344.4	27.13	7.52	4.15	0.66	1.77
13.54977	3.713677	478.3	5.85	1.83	2.45	0.87	1.88
10.49095	5.024514	361.7	4.82	1.11	3.35	1	2.99
7.60181	7.469324	359.4	10.13	3.49	3.63	0.67	2.86
6.807692	8.357201	332.1	19.52	2.3	2.82	0.61	2.58
5.363122	9.965055	464.3	3.73	0.98	3.35	1.66	2.2
9.295249	5.161416	377.4	5.25	1.7	3.18	1.04	2.29
7.426471	6.355704	352	5.72	0.76	3.2	1.46	2.37
11.12557	3.174551	394.7	7.08	1.48	3.1	0.92	2.58
10.60064	5.352437	442.5815	7.572593	1.981111	3.15	1.012593	2.375926
2.836239	1.814352	111.4098	5.392831	1.619837	0.629847	0.362031	0.58818
