# Supplementary figures and images for: Serum Biomarkers for Chronic Renal Failure Screening and Mechanistic Understanding: A Global LC-MS-Based Metabolomics Research
Source: Evid Based Complement Alternat Med. 2022 Jul 30;2022:7450977. doi: 10.1155/2022/7450977 (PMC9356786; doi:10.1155/2022/7450977)

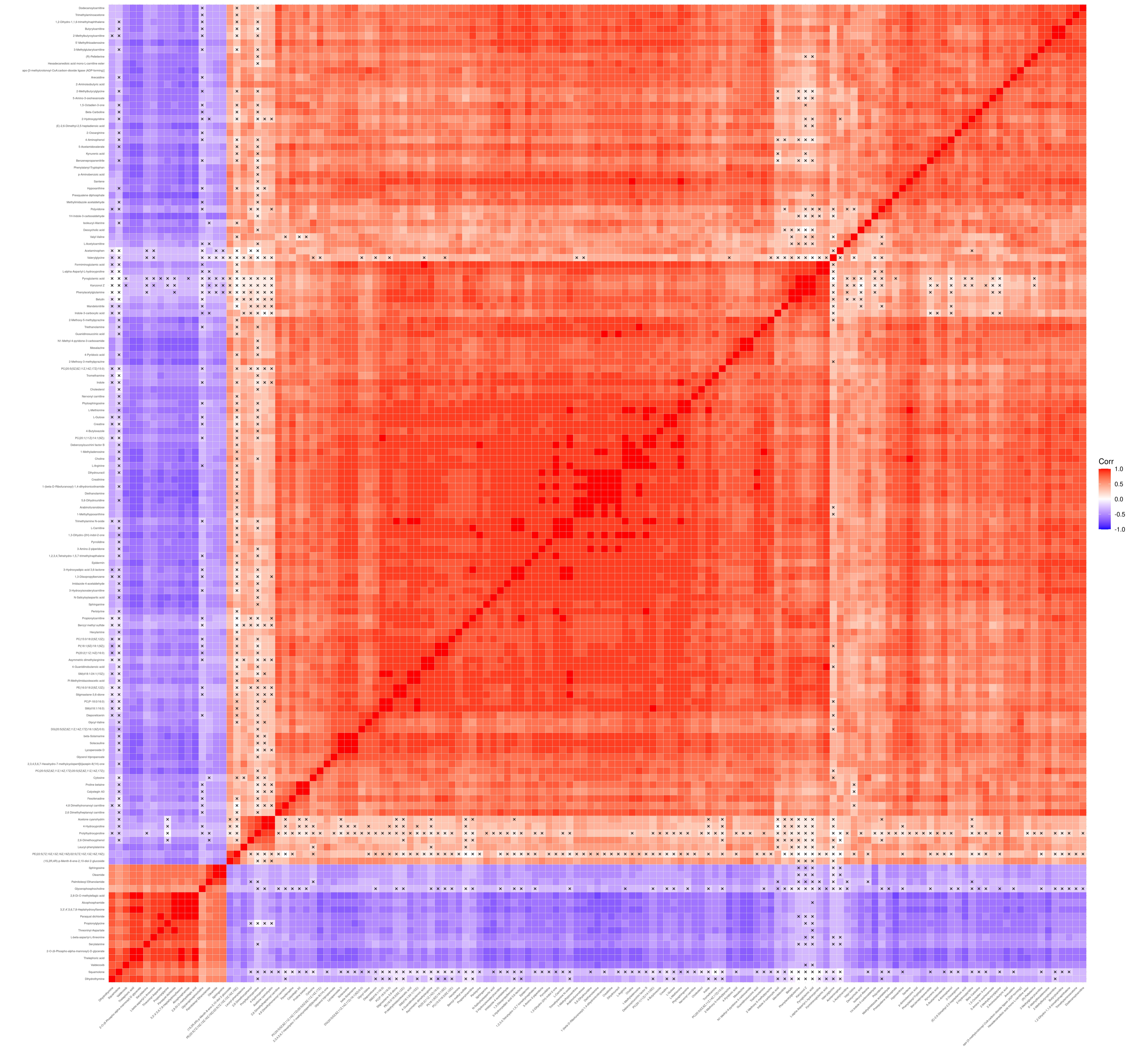

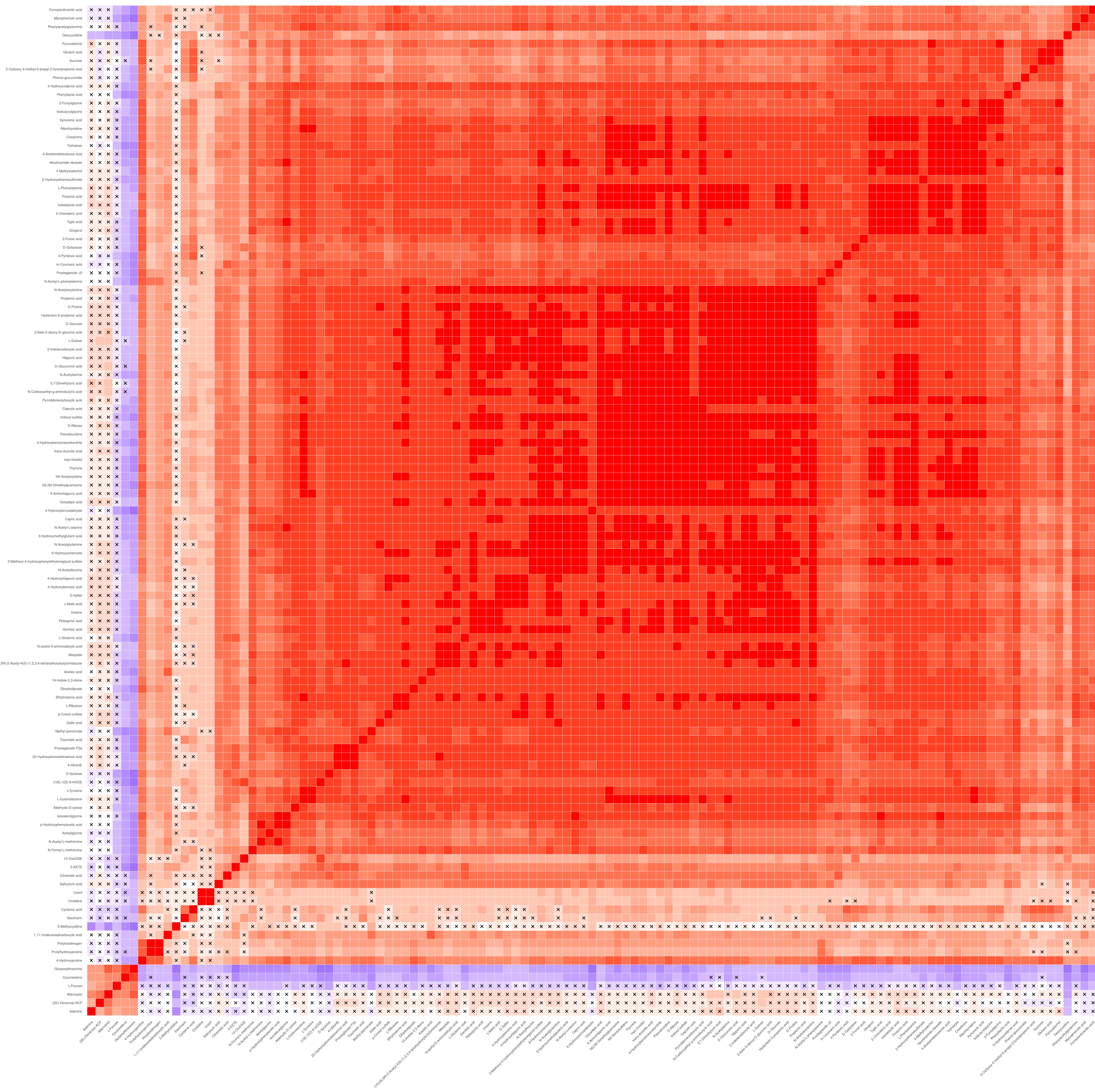

Supplement: Supplementary Materials — Supplementary Figure S1: Quality control diagram. A and B are TIC diagrams of QC samples; C and D are EIC diagrams of internal standard in QC sample; E and F are PCA analysis of QC samples. Supplementary Figure S2: Correlation analysis heat map in positive and negative modes, respectively. Supplementary Figure S3: Dot map of all the different endogenous metabolites. Supplementary Figure S4: Heatmap of hierarchical clustering analysis of group RF vs HC. Supplementary Figure S5: Metabolic pathways with red/blue dots representing the differentially expressed compounds. Red means up regulation, blue means down regulation. Supplementary Figure S6: a KEGG metabolic pathway, Arginine and Proline metabolism. Supplementary Figure S6: b KEGG metabolic pathway, Sphingolipid metabolism. Supplementary Figure S6: c KEGG metabolic pathway, Glycerophospholipid metabolism. Supplementary Figure S6: d KEGG metabolic pathway, D-Arginine and D-ornithine metabolism. Supplementary Figure S7: a KEGG metabolic pathway, Phenylalanine metabolism. Supplementary Figure S7: b KEGG metabolic pathway, Ascorbate and aldarate metabolism. Supplementary Figure S7: c KEGG metabolic pathway, D-Glutamine and D-glutamate metabolism. Supplementary Figure S7: d KEGG metabolic pathway, Arginine and proline metabolism. Supplementary Figure S8: The typical mass spectra of metabolites. Supplementary Table S1: The clinical characteristics of patients. Supplementary Table S2: POS-Differentially Expressed Metabolites. Supplementary Table S3: NEG-Differentially Expressed Metabolites. [file 7450977.f1.zip › Supplementary Figure S2.pdf]

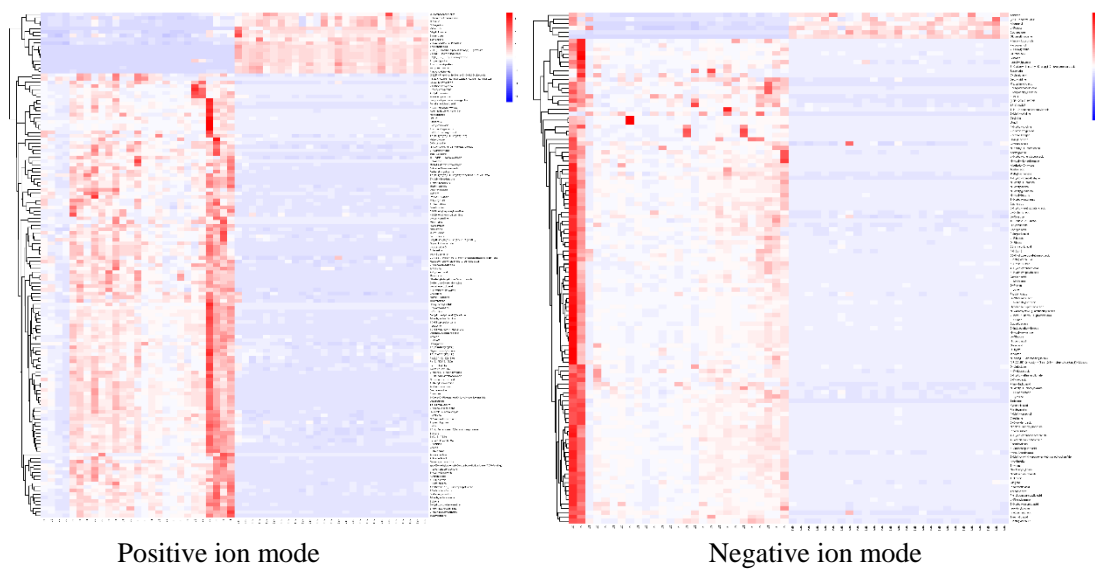

**Supplementary Figure S4** Heatmap of hierarchical clustering analysis of group RF vs HC.

Supplement: Supplementary Materials — Supplementary Figure S1: Quality control diagram. A and B are TIC diagrams of QC samples; C and D are EIC diagrams of internal standard in QC sample; E and F are PCA analysis of QC samples. Supplementary Figure S2: Correlation analysis heat map in positive and negative modes, respectively. Supplementary Figure S3: Dot map of all the different endogenous metabolites. Supplementary Figure S4: Heatmap of hierarchical clustering analysis of group RF vs HC. Supplementary Figure S5: Metabolic pathways with red/blue dots representing the differentially expressed compounds. Red means up regulation, blue means down regulation. Supplementary Figure S6: a KEGG metabolic pathway, Arginine and Proline metabolism. Supplementary Figure S6: b KEGG metabolic pathway, Sphingolipid metabolism. Supplementary Figure S6: c KEGG metabolic pathway, Glycerophospholipid metabolism. Supplementary Figure S6: d KEGG metabolic pathway, D-Arginine and D-ornithine metabolism. Supplementary Figure S7: a KEGG metabolic pathway, Phenylalanine metabolism. Supplementary Figure S7: b KEGG metabolic pathway, Ascorbate and aldarate metabolism. Supplementary Figure S7: c KEGG metabolic pathway, D-Glutamine and D-glutamate metabolism. Supplementary Figure S7: d KEGG metabolic pathway, Arginine and proline metabolism. Supplementary Figure S8: The typical mass spectra of metabolites. Supplementary Table S1: The clinical characteristics of patients. Supplementary Table S2: POS-Differentially Expressed Metabolites. Supplementary Table S3: NEG-Differentially Expressed Metabolites. [file 7450977.f1.zip › Supplementary Figure S4.pdf]
